# Proteomic Profiling of *Plasmodium* Sporozoite Maturation Identifies New Proteins Essential for Parasite Development and Infectivity

**DOI:** 10.1371/journal.ppat.1000195

**Published:** 2008-10-31

**Authors:** Edwin Lasonder, Chris J. Janse, Geert-Jan van Gemert, Gunnar R. Mair, Adriaan M. W. Vermunt, Bruno G. Douradinha, Vera van Noort, Martijn A. Huynen, Adrian J. F. Luty, Hans Kroeze, Shahid M. Khan, Robert W. Sauerwein, Andrew P. Waters, Matthias Mann, Hendrik G. Stunnenberg

**Affiliations:** 1 Department of Molecular Biology, NCMLS, Radboud University Nijmegen, Nijmegen, The Netherlands; 2 Center for Molecular and Biomolecular Informatics, NCMLS, Radboud University Nijmegen Medical Centre, Nijmegen, The Netherlands; 3 Leiden Malaria Research Group, Department of Parasitology, Centre for Infectious Diseases, Leiden University Medical Center, Leiden, The Netherlands; 4 Department of Medical Microbiology, NCMLS, Radboud University Nijmegen Medical Centre, Nijmegen, The Netherlands; 5 Center for Experimental BioInformatics, Department of Biochemistry and Molecular Biology, University of Southern Denmark, Odense, Denmark; Washington University School of Medicine, United States of America

## Abstract

*Plasmodium falciparum* sporozoites that develop and mature inside an *Anopheles* mosquito initiate a malaria infection in humans. Here we report the first proteomic comparison of different parasite stages from the mosquito—early and late oocysts containing midgut sporozoites, and the mature, infectious salivary gland sporozoites. Despite the morphological similarity between midgut and salivary gland sporozoites, their proteomes are markedly different, in agreement with their increase in hepatocyte infectivity. The different sporozoite proteomes contain a large number of stage specific proteins whose annotation suggest an involvement in sporozoite maturation, motility, infection of the human host and associated metabolic adjustments. Analyses of proteins identified in the *P. falciparum* sporozoite proteomes by orthologous gene disruption in the rodent malaria parasite, *P. berghei*, revealed three previously uncharacterized *Plasmodium* proteins that appear to be essential for sporozoite development at distinct points of maturation in the mosquito. This study sheds light on the development and maturation of the malaria parasite in an *Anopheles* mosquito and also identifies proteins that may be essential for sporozoite infectivity to humans.

## Author Summary

Human malaria is caused by *Plasmodium falciparum*, a unicellular protozoan parasite that is transmitted by *Anopheles* mosquitoes. An infectious mosquito injects saliva containing sporozoite forms of the parasite and these then migrate from the skin to the liver, where they establish an infection. Many intervention strategies are currently focused on preventing the establishment of infection by sporozoites. Clearly, an understanding of the biology of the sporozoite is essential for developing new intervention strategies. Sporozoites are produced within the oocyst, located on the outside wall of the mosquito midgut, and migrate after release from the oocysts to the salivary glands where they are stored as mature infectious forms. Comparison of the proteomes of sporozoites derived from either the oocyst or from the salivary gland reveals remarkable differences in the protein content of these stages despite their similar morphology. The changes in protein content reflect the very specific preparations the sporozoites make in order to establish an infection of the liver. Analysis of the function of several previously uncharacterized, conserved proteins revealed proteins essential for sporozoite development at distinct points of their maturation.

## Introduction

The life cycle of human malaria parasite *Plasmodium falciparum* within the mosquito vector begins when gametocytes are taken up in an infected blood meal; after forming gametes and fertilisation, the resulting zygote differentiates into a motile ookinete that traverses the midgut epithelium and transforms within 36–48 hours into an oocyst (OOC) between the midgut epithelial cells and the basal lamina. The oocyst is an asexually replicating form of the parasite, which produces up to 2000–4000 sporozoites in about two weeks. Rupture of mature oocysts releases oocyst-derived sporozoites (ODS) into the hemocoel of the mosquito. The movement of the hemolymph brings the ODS in contact with the salivary glands, which they then invade. The sporozoites mature inside the salivary glands and then are stored ready for transmission to the mammalian host upon the next blood meal. A limited number of the salivary gland sporozoites (SGS) are injected during a mosquito bite and only a few of these complete the necessary migration from the skin to the liver to establish an infection inside hepatocytes. Clearly, the sporozoite has to complete a number of functions and metabolic readjustments both before and after injection into a mammalian host. The sporozoite has to be capable of actively exiting an oocyst, travelling through the hemolymph (the mosquito circulatory system), and invading salivary glands. Further, following a mosquito bite injection the sporozoites enters a very different physiological environment of the human host, and then has to traverse through human endothelial cells, possibly Kupffer cells and finally hepatocytes where they establish an infection; moving all the time using a specialized form of gliding motility. Despite all these events the general morphology of the sporozoite is not visibly altered at any stage (for general reviews on sporozoite biology please see the following references and the references therein [1]–[7]).

Since the sporozoite plays an essential role in the first phase of a malaria infection, an understanding of its biology is of great importance in order to develop intervention methods against initial infection and consequently disease. A wealth of gene expression data from high throughput studies exists on the intracellular erythrocytic growth and development of *Plasmodium* parasites [8]–[17], whereas far less is known about the genes/proteins involved in sporozoite development [10], [11], [16], [18]–[22]. Indeed, only a few (less than 25) proteins have been characterized as being essential for sporozoite development and infectivity. These include several proteins that are currently under investigation as either potential subunit vaccines (such as circumsporozoite protein (CS) and thrombospondin related anonymous protein (TRAP)) or may serve in the generation of whole organism, genetically attenuated sporozoite vaccines [23]–[26] when the genes encoding these proteins are eliminated from the *Plasmodium* genome, such as UIS3, UIS4 [27],[28] and P36 and P36p [29],[30]. The lack of large scale *in vitro* culture methods for oocysts and sporozoites has restricted high throughput protein expression studies to only mature sporozoites, which are more readily obtained from infected salivary glands.

In this study we have performed a detailed proteomic comparison of sporozoites obtained from both oocysts and salivary glands which were obtained by hand-dissection of infected mosquito midguts and salivary glands. The proteome analysis was performed using essentially the same high throughput mass spectrometric analysis that we previously applied to generate the proteomes of the blood stages of *P. falciparum* [15] as well as the proteomes of male and female gametocytes of *P. berghei* [14]. Our analyses resulted in a proteome of oocysts (n = 127), oocyst-derived sporozoites (n = 450) and salivary gland sporozoites (n = 477), which represent 728 individual *Plasmodium* proteins, of which 250 were exclusively detected in the oocyst/sporozoite stages when compared to the *P. falciparum* blood stage proteomes generated in a previous study [15]. The identification of proteins and their relative distributions within the different proteomes suggest specific metabolic adaptations and other biological functions of the maturing sporozoite. Moreover, we analyzed the function of eight sporozoite-specific proteins identified in our proteome analyses that were specifically annotated as hypothetical proteins, by targeted gene disruption of the orthologous genes of the rodent malaria parasite, *P. berghei*. We were able to demonstrate an essential and distinct role for three of these proteins in sporozoite development.

## Results

### Mosquito stage proteome

**Figure 1:**
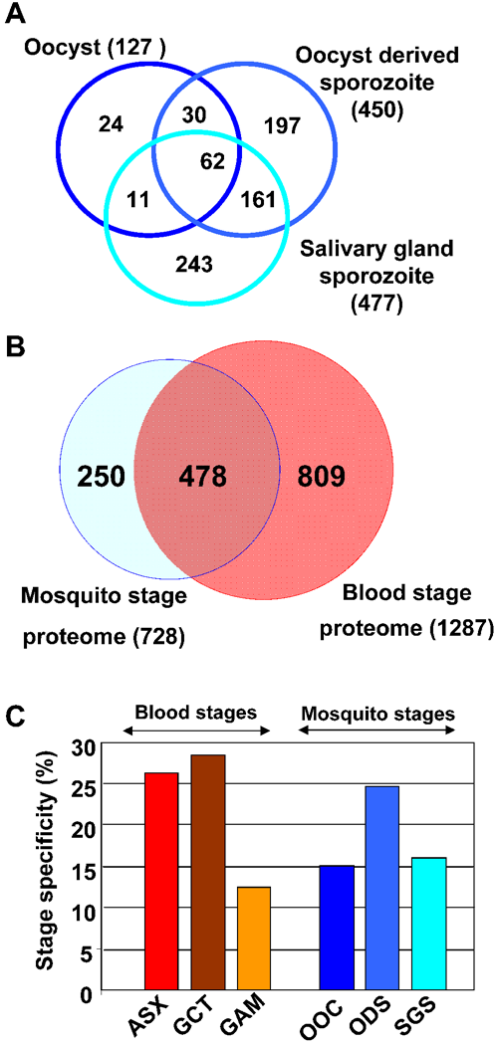
Distribution of identified *P. falciparum* proteins over different life-cycle stages. (A) Venn diagram depicting the distribution of detected *P. falciparum* proteins over three different mosquito life-cycle stages (oocysts, oocyst-derived sporozoites and salivary gland sporozoites). Numbers represent the number of proteins, that are either shared between 2 or 3 stages (overlapping areas) or that are detected in a single stage. (B) Comparison of the expression of *P. falciparum* proteins detected in the three mosquito stage proteomes to the blood stage proteomes described previously [15]. (C) The percentage of proteins exclusively detected in only one proteome out of 6 different life cycle stage proteomes, i.e. ASX - asexual blood stages; GCT – gametocytes; GAM – gametes; OOC – oocysts; ODS - oocyst-derived sporozoites; SGS - salivary gland sporozoites.

Protein samples derived from infected mosquito midguts and salivary glands were analyzed by nano–liquid chromatography tandem mass spectrometry (nLC-MS/MS) essentially as previously described [15]. The MS/MS spectra were searched against a combined database of all possible predicted tryptic peptides derived from all *P. falciparum*, human, and mosquito (*Anopheles gambiae*) proteins. The proteomic analysis of *P. falciparum* oocysts, oocyst-derived sporozoites, and salivary gland sporozoites resulted in a total of 4611 unique peptides mapping to 728 non redundant *P. falciparum* proteins; they are distributed over the three stages with 127, 450 and 477, respectively and depicted as a Venn diagram in Figure 1A. Identified tryptic peptides and corresponding *Plasmodium* proteins of the mosquito stages are provided as supplementary material (Table S1). In our previous analysis of infected human red blood cells we identified 741 asexual blood stage parasite proteins from a mixture of schizonts and trophozoites and an additional 931 gametocyte and 645 gamete proteins [15]. Merging these datasets with the proteomes of the mosquito stages resulted in the identification of 250 *Plasmodium* proteins (Table S1) that are specifically detected in mosquito stages and 809 proteins that are expressed only in the blood stages (Figure 1B). However, it is important to note that due to the incomplete nature of all proteome datasets, absence of proteins from one dataset may also be due to the limits of detection and not the actual absence of expression. Parasite samples derived from infected mosquitoes were considerably contaminated with mosquito proteins with total parasite protein fractions of 35% for ODS, 31% for SGS and for OOC only 11% of the sequenced proteins were parasite in origin. Therefore this relatively high degree of contamination resulted in overall lower numbers of proteins compared to our previous *Plasmodium* infected blood stage proteome study. In particular, only 127 *P. falciparum* proteins in a pool of 987 mosquito proteins were identified for the oocyst sample that presumably represents the more abundantly expressed parasite proteins. Therefore, further analysis of the identified proteins and additional functional analyses are mainly focused on the proteins identified in the ODS and SGS. In total, we analyzed six different stages of *Plasmodium* (both from this study and our previous work) and have identified a total of 1543 *Plasmodium* proteins. The proportion of ‘stage specific’ proteins in the different life cycle stages ranged from 12% (gametes) to 28% and the stage specificity of proteins in the mosquito stages ranged between 15–24% (Figure 1C).

### Comparison with existing RNA/protein mosquito stage studies

Genome-wide proteome and transcriptome studies have previously been reported for salivary gland sporozoites of *P. falciparum* [10],[16], for oocysts and sporozoites of *P. berghei* [11] and recently for oocyst-derived sporozoites and salivary gland sporozoites of *P.yoelii* [22]. The Florens et al SGS proteome [10] identified a total of 1048 proteins of which 314 proteins include at least one peptide that is fully tryptic. It has been shown that selection of only fully tryptic peptides greatly increases the confidence in each protein within the proteome and was similarly applied to our dataset [31]. Comparison of these ‘fully-tryptic proteins’ (proteins identified by peptides conforming to proper tryptic cleavage) with the ‘fully-tryptic proteins’ from our SGS proteome (n = 477) shows that 166 proteins are present in both proteomes (i.e. 53% of the Florens' data (Table S2)). Moreover, in order to further increase our confidence in the ‘protein-calling’ in both datasets, a comparison was made using only those proteins that were identified by 2 or more fully-tryptic peptides (i.e. 346 proteins from our mosquito stage proteome and 82 from the Florens SGS proteome). In this analysis, we found that 72 proteins were in common (i.e. 88% of the Florens enriched SGS proteome). Interestingly, we fail to find any PfEMP-1 proteins, as had previously been reported in the Florens et al SGS proteome, in either dataset when we examine only the “fully-tryptic peptide proteomes” [10].

The oocyst proteome of *P. berghei* described by Hall et al [11] detected of a total of 220 proteins of which 175 proteins have an orthologue in *P. falciparum* and 87 of these (i.e. 50%) were also detected in our mosquito proteomes (Table S2). Again consideration of only fully tryptic peptides revealed that 60 of the resulting 111 *P. berghei* orthologs (i.e. 54%) were found in common. Similarly, of the 108 proteins identified in the *P. berghei* SGS proteome 86 proteins have an orthologue in *P. falciparum* (Table S2) of which 46 (i.e. 53% of the Hall SGS proteome) were detected in our SGS proteome of *P. falciparum*. There were only 20 fully-tryptic proteins in the Hall SGS proteome of which 75% (n = 15) were also detected in our *P. falciparum* SGS proteome. Selecting the 202 genes that were commonly expressed in our SGS proteome and in the published SGS proteome of *P. falciparum* [10], the relative abundance of protein in the two datasets was examined using a Pearson correlation. The emPAI peptide counting method using the number of observed peptides detected per protein and corrected to the number of expected tryptic peptides was applied to compute relative protein levels [32],[33]. A good correlation (r = 0.73) existed between protein abundance levels (emPAI values; see Materials and Methods section) in our SGS proteome and the previous *P. falciparum* SGS proteome.

However, when we compared abundance of our SGS proteins (i.e. by emPAI values) with the abundance of mRNA SGS transcripts reported by Le Roch and Zhou et al [16],[22] we found a lower correlation value (i.e. 0.31 and 0.33 respectively (Table S3)).

Several (smaller scale) studies have been reported that using either subtractive hybridization or cDNA quantification methods (i.e. Serial Analysis of Gene Expression (SAGE)) to identify sets of genes transcribed in sporozoites in the rodent malaria parasites, *P. berghei* [20],[21] and *P. yoelii* [18]. Comparison of the identified *P. yoelii* mRNAs with our proteomes showed that for nearly all genes transcribed in sporozoites (20 out of 23 sporozoite (S) genes), proteins were detected in our sporozoite proteomes (Table S4). This may suggest that for a significant proportion of genes transcription and protein expression coincide within the sporozoite. However, a weaker correlation was found between transcription in *P. berghei* sporozoites and the presence of protein in our proteomes. Specifically, we were able to detect protein for 34 of the 98 genes identified in the *P. berghei* sporozoites SAGE analysis (i.e. the Sporozoite expressed gene Identified by SAGE (SIS) genes (Table S4)) but only 5 out of 26 transcribed genes in the Suppression Subtractive Hybridization (SSH) analysis (i.e. the Upregulated In Sporozoites (UIS) genes (Table S4)). It is however interesting to observe that between the two SSH studies only 2 out of 30 genes appear clearly up-regulated in both *P. yoelii* and *P. berghei* sporozoites.

### Functional annotation of mosquito stage proteins

**Figure 2:**
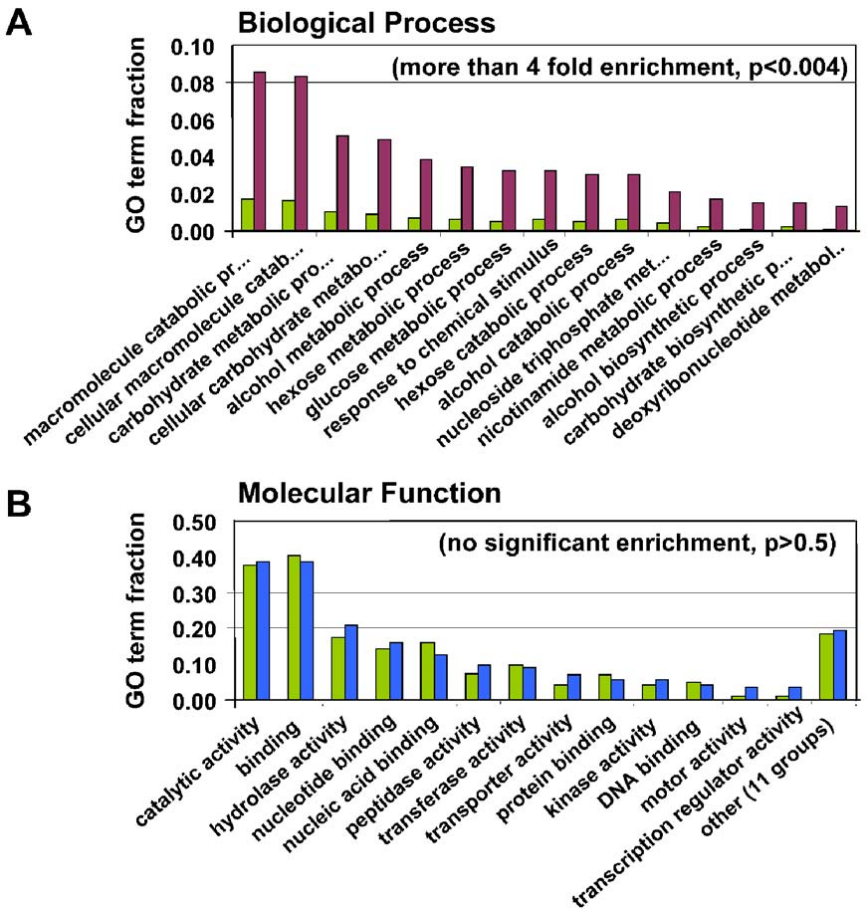
Gene Ontology term enrichment analysis of mosquito stage proteome. (A) Enrichment for GO ‘Biological Process’ terms of proteins detected in mosquito and blood stages. The figure shows terms on the x-axis that are significantly enriched (p<0.004) by more than four fold. GO terms of the shared set of proteins (n = 478, purple bars) is compared to terms of all predicted *P. falciparum* proteins (5410, green bars). The y-axis displays the fraction relative to all GO Biological Process terms. (B) Enrichment for GO ‘Molecular Function’ main terms of proteins detected specifically in mosquito stages (and blood stages). GO terms of the mosquito specific set of proteins (n = 250, blue bars) is compared terms of all predicted *P. falciparum* proteins (5410, green bars). The y-axis displays the fraction relative to all GO Molecular Function terms. These terms do not show a significant enrichment (p>0.5).

A global functional characterization of the ‘mosquito stage’ proteome was performed by an enrichment analysis of Gene Ontology (GO) annotations, for both the proteins that are shared between blood stages and mosquito stages (n = 478) and for the mosquito stage specific proteins (n = 250). The set of 478 genes commonly expressed in both mosquito and blood stages showed enrichment in GO annotations in all classes (i.e. Molecular Function, Cellular Component and Biological process (Figure S1)) and this enrichment is principally associated with housekeeping genes (Figure 2). The mosquito stage specific proteome did not reveal significant (p<0.01) enrichment in GO annotations nor did additional analyses for GO enrichment of the mosquito stage specific proteins using BINGO [34] and Ontologizer [35] (data not shown). In Figure 2B GO categories (Molecular Function) are shown for the mosquito stage specific proteome that contain more than 5 proteins. The lack of enrichment could be caused by the high proportion of genes annotated as hypothetical (300 out of 728) and consequently the relatively large number of proteins in the mosquito stage specific proteome (124 out of 250) without a GO annotation. Since our analysis did not reveal a significant GO enrichment for proteins known to be important in sporozoite function (e.g. motility and motor activity (Figure 2)) we analyzed our mosquito stage proteome for previously reported proteins, for which a function during sporozoite development is described and supported by strong experimental evidence (e.g. gene-knockout and/or antibody-inhibition studies). These proteins, in total 23, are listed in Table 1 and 15 out of 23 proteins are present in the mosquito stage proteome reported here. Based on a total number of 5410 genes in the genome of *P. falciparum* and 728 proteins in our mosquito stage proteome, these 15 proteins represent a 4.8 fold functional enrichment relative to the annotated genome and is highly significant (p<0.001 using Ontologizer). A good agreement exists between the function of the sporozoite proteins as shown in Table 1 and their expression pattern in the different mosquito stages. For example, proteins with multiple roles during sporozoite maturation (e.g. CS and TRAP) were identified in all stages (OOC, ODS and SGS) whereas proteins involved in hepatocyte traversal, such as SPECT1, SPECT2 (sporozoite microneme protein essential for cell traversal 1 and 2) and CelTOS (cell-traversal protein for ookinetes and sporozoites) were exclusively identified in mature SGS.

**Table 1:** Characterized proteins involved in sporozoite development and invasion of host cells.

Accession nr	protein name (1)	protein involved in (2)	nr unique pept/protein in life cycle stages (3)						Reference
			ASX	GCT	GAM	OOC	ODS	SGS	
PF14_0067	CCp3	sporozoite development	-	31	10	-	-	-	Claudianos [72], Pradel [73]
PF14_0532	CCp2	sporozoite development	-	39	20	-	-	-	Pradel [73]
PFC0180c	IMC1	sporozoite development (cell shape)	-	-	-	0	9	25	Khater [74]
PFC0210c	CS	sporozoite development, salivary gland and hepatocyte invasion	-	-	-	2	5	9	Menard [43], Wang [44]
PFB0325c	cysteine protease	egress from oocyst	-	-	-	-	16	23	Aly [56]
PF13_0233	myosin A	sporozoite gliding motility	-	-	-	2	39	49	Bergman [75]
PFL2225w	MTIP	sporozoite gliding motility	1	1	-	2	4	3	Bergman [75]
PF13_0201	SSP2/TRAP	sporozoite gliding motility, salivary gland and hepatocyte invasion	-	-	-	1	6	35	Rogers [45], Sultan [46]
PFI0550w	CRMP1	salivary gland invasion	-	1	-	-	-	-	Thompson [76]
MAL7P1.92	CRMP2	salivary gland invasion	-	-	-	-	-	-	Thompson [76]
PF11_0486	MAEBL	salivary gland invasion	-	-	-	-	33	1	Kariu [42]
MAL13P1.212	SPECT1	cell traversal hepatocytes	1	-	-	-	-	16	Ishino [77]
PFD0430c	SPECT2	cell traversal hepatocytes	1	-	-	-	-	26	Ishino [78]
PFL0800c	CelTOS	cell traversal hepatocytes	-	-	-	-	-	6	Kariu [79]
PFF1420w	PL	cell traversal hepatocytes	-	-	-	-	-	-	Bhanot [80]
PF11_0344	AMA-1	hepatocyte invasion	1	-	-	-	2	19	Silvie [81]
PFB0570w	SPATR	hepatocyte invasion	-	-	-	-	-	5	Chattopadhyay [82]
PFB0095c	PfEMP3	hepatocyte invasion	3	-	-	-	-	-	Grüner [83]
PF07_0006	STARP	hepatocyte invasion	-	-	-	-	-	-	Pasquetto [84]
GI:1477963	SALSA	hepatocyte invasion	-	-	-	-	-	-	Puentes [85]
PFA0200w	TRSP	hepatocyte invasion	-	-	-	-	1	3	Labaeid [86]
PFD0215c	pf52 protein/P36p	hepatocyte invasion - development	-	-	-	-	-	5	Ishino [29], van Dijk [30]
PFD0210c	pbs36 homologue/P36	hepatocyte invasion - development	-	-	-	-	-	8	Ishino [29]

1 CCP (members of the LCCL protein family), IMC (inner membrane complex), CS (circumsporozoite), MTIP (myosin A tail domain interacting protein), SSP2 (sporozoite surface protein), TRAP (Thrombospondin related anonymous protein), CRMP (cysteine repeat modular protein), MAEBL (membrane antigen erythrocyte binding like protein ), SPECT (sporozoite microneme protein essential for cell traversal ), CelTOS (cell-traversal protein for ookinetes and sporozoites), PL (phosphoplipase), AMA (apical membrane antigen), SPATR (secreted protein with altered thrombospondin domain), EMP (erythrocyte membrane protein), STARP (sporozoite threonine and asparagine-rich protein), SALSA (sporozoite and liver stage antigen); TRSP (thrombospondin related protein).

2 The role of these proteins has been determined by functional analysis in gene-knockout and/or antibody-inhibition studies.

3 ASX - asexual blood stages; GCT – gametocytes; GAM – gametes; OOC – oocysts; ODS - oocyst-derived sporozoites; SGS - salivary gland sporozoites.

Sporozoites, like other motile stages (except male gametes) of Apicomplexan organisms, move on substrates by a mechanism known as gliding motility which is driven by an actomyosin motor complex [3],[36],[37]. Although there was no enrichment with high confidence (p<0.05) of the GO Molecular Function category ‘motor activity’ for mosquito stage specific proteins (Figure 2), several proteins known to be involved in the actomyosin motor complex are well represented and include TRAP, myosin A, MyoA Tail Domain Interacting Protein (MTIP), actin and F-1,6-BP aldolase (3.6 fold enrichment with low confidence). Additionally, sporozoites encode a variety of surface molecules for both motility and invasion of host cells. For apicomplexan parasites members of the TRAP/MIC2 family have been shown to be important for host cell recognition and motility. The general architecture of this family is typified by one or more thrombospondin type I (TSP1) domains in their extracellular regions which may in addition also posses von Willebrand factor A (vWA) extracellular domains [38]. Our sporozoite proteome shows a 4.7 fold enrichment for proteins that contain one or multiple TSP1 domains (Table 2) compared to the *P. falciparum* proteome of 5410 proteins.

**Table 2:** Expression of *Plasmodium* proteins containing thrombospondin type 1 (TSP1) and/or von Willebrand factor A (vWA) domains in different life cycle stage proteomes.

Accession nr	Protein name	domain		nr unique pept/prot in life cycle stages (1)						Reference
		1	2	ASX	GCT	GAM	OOC	ODS	SGS	
PF13_0201	TRAP	TSP1	VWA	-	-	-	**1**	**6**	**35**	Rogers [45], Sultan [46]
PFC0210c	CS	TSP1		-	-	-	**2**	**5**	**9**	Menard [43], Wang [44]
PFA0200w	TRSP	TSP1		-	-	-	-	**1**	**3**	Kaiser [18], Labaeid [86]
MAL8P1.45	hypothetical protein	TSP1		-	-	-	-	**1**	-	Aravind [38]
PFB0570w	SPATR	TSP1							**5**	Chattopadhyay [82]
PFF0800w	TRAP-like protein	TSP1	VWA	-	**1**	-	-	-	**2**	Moreira [87]
PFL0870w	PTRAMP	TSP1		-	-	-	-	-	**1**	Thompson [76]
PF10_0281	MTRAP	TSP1		-	-	-	-	-	-	Baker [88]
PF08_0136b	WARP	TSP1		-	-	-	-	-	-	Yuda [89]
PFC0640w	CTRP	TSP1	VWA	**1**	**2**	-	-	-	-	Baker [88]
PFL0875w	hypothetical protein	TSP1		-	-	-	-	-	-	Aravind [38]

1 ASX - asexual blood stages; GCT – gametocytes; GAM – gametes; OOC – oocysts; ODS - oocyst-derived sporozoites; SGS - salivary gland sporozoites.

### Two sporozoite proteomes – ODS versus SGS

Although the morphology of oocyst-derived and salivary gland sporozoites is identical at the level of light microscopy, ODS of *P. berghei* are significantly less infective to the mammalian host than SGS [39]. This marked difference in infectivity suggests significant developmental changes between these forms and was indicated by the analyses of gene transcription of different sporozoite stages by either SSH screens or SAGE analysis, which alludes to changes in protein expression in the sporozoite during the period of egress from the oocysts and the establishment of infection of the salivary glands [18],[20],[21]. In agreement with these observations, we found a large number of proteins expressed in SGS that were absent or relatively low expressed in ODS (Table S1). Several proteins involved in metabolic pathways show clear differences in distribution between ODS and SGS (Figure S2). For example, 8 out of 9 enzymes of the glycolytic pathway for ATP production were detected, all which were either more abundant or exclusive to SGS (SGS 8 proteins with 140 peptides; ODS 4 proteins and 48 peptides). A similar profile is observed for proteins involved in the production of NADPH via the pentose phosphate pathway with an up-regulation of these proteins in SGS (5 proteins and 26 peptides) compared to ODS (1 protein and 2 peptides). A third up-regulated metabolic pathway is the tricarboxylic acid (TCA) cycle (7 proteins and 85 peptides in SGS compared to 4 proteins and 26 peptides in ODS). Interestingly, several genes (4 out of 10) of the TCA cycle are most abundantly expressed in SGS, not only when compared to ODS but also in comparison with the blood stages, indicating an important role of the TCA cycle in mature sporozoites. Also the enzyme phosphoenolpyruvate carboxykinase (PF13_0234) is upregulated in the salivary gland sporozoites (8 peptides in ODS and 17 in SGS), which is again in agreement with the upregulation of enzymes involved in the TCA cycle and glycolysis [40]. It also appears that SGS prepare for enhanced protein synthesis: 9 of the 11 detected tRNA ligases are only detected in the SGS proteome and not in the ODS proteome (Table S1) as are ribosomal proteins, translation elongation factors and the TCP chaperonin complex proteins, which are either exclusively detected in SGS or are represented in the SGS proteome by substantially more peptides compared to the ODS proteome. As is shown in Table 1, proteins that are known to play a role in traversal and invasion of hepatocytes are highly enriched in SGS. On the other hand, the expression of MAEBL that is expressed along with CS and well before AMA-1 [41] and is known to function in attachment and invasion of the salivary gland [42] is more abundantly expressed in ODS. Therefore, it would appear that the proteomes of the sporozoite characterised by this study at different stages of development accurately reflect the functionality of either the ODS or SGS.

Consequently, based on the expression pattern and relative abundance of the peptides in the proteomes from OOC, ODS and SGS (see Materials and Methods section) the mosquito stage specific proteins can be regarded as belonging to one of 3 distinct groups (Table S1): Group I consists of 112 ODS proteins highly enriched for the ODS stage, putatively involved in sporozoite maturation inside the oocyst and in salivary gland invasion; similarly Group II which contains 74 proteins up-regulated in SGS potentially involved in infection of the mammalian host; and finally Group III that contains 59 proteins that are shared between the different mosquito stage proteomes and therefore may be involved in sporozoite functions necessary both in the mosquito vector and the mammalian host (e.g. proteins involved in gliding motility and invasion such as CS [43],[44] and TRAP [45],[46] (Table 1)). These three groups formed the basis for selection of genes for further functional analysis of their encoded proteins through targeted disruption of the orthologous genes in the rodent malaria parasite, *P. berghei*. The three groups were further refined for subsequent functional analysis using the following criteria (see also Materials and Methods section): i) high expression level as determined by the number of uniquely detected peptides per protein, ii) presence of gene sequences encoding putative transmembrane regions, signal peptides and/or GPI anchors, and iii) presence exclusively in the mosquito stage proteomes. This resulted in selection of genes as shown in Table 3. Further, in order to enrich for proteins that may define *Plasmodium* specific functions, we preferentially selected not only genes that were annotated as hypothetical but also had no domains predicted by either the SMART or Pfam algorithms (i.e. with no indication of predicted function).

**Table 3:** Characteristics of mosquito stage specific proteins selected on the basis of expression pattern during sporozoite development.

Accession nr (1)	Protein Name	MW (kDa)	Group (2)	MOTIF PREDICTION (3)					ORTHOLOGY PREDICTION (4)				nr unique peptide/protein (5)			
				SP	GPI	nr TM (mean)	PEXEL	SMART domain	Ortho MCL group	nr species	additional nr species		OOC	ODS	SGS	Mosq. fr.
										Plasm.	Api	other				
**PF11_0528**	**hypothetical protein**	710	I			5.3			OG2_80072	6	1	0	1	32		0.97
PF11_0486	MAEBL	243	I	1		1.3			OG2_70757	6	3	>10		33	1	1
MAL13P1.66	hypothetical protein	346	I			1.8			OG2_93592	6	0	0		26	1	0.93
PFF0645c	integral membrane protein	163	I			6.5		DUF1222	OG2_76677	6	3	9		18	1	0.95
PF14_0404	hypothetical protein	408	I			0.8			OG2_71079	6	1	>10		20		0.91
PF14_0722	CRMP4	699	I	1		9.0		3 * GCC2_GCC3	OG2_81006	5	0	0		16		0.94
**PF14_0435**	**hypothetical protein**	144	I	1		6.8			OG2_89567	5	0	0		13		1
PF13_0338	hypothetical protein (Pf92)	93	I	1	1	1.0		s48-s45	OG2_117922	3	0	0		11		1
MAL8P1.126	serine protease	101	I	1		1.5	1	Trypsin	OG2_77036	3	3	>10		11		1
**PF14_0607**	**hypothetical protein**	127	I	1		4.8	1		OG2_79434	6	5	0		9		1
MAL8P1.135	hypothetical protein	116	I			8.8			OG2_74819	6	2	>10		8		1
PF14_0694	protein disulfide isomerase	66	I	1		0.8		Thioredoxin	OG2_78996	6	5	3		8		1
PF11_0229	hypothetical protein	57	I	1	1	0.8			OG2_93024	6	0	0		7		1
PFE0340c	hypothetical protein	87	I			5.0		Rhomboid	OG2_70960	6	5	>10		6		1
MAL13P1.119	cAMP-specific 3′,5′-cyclic	86	I			4.5		HDc	OG2_93460	6	0	0		6		1
	phosphodiesterase 4B															
PFD0295c	hypothetical protein	85	I	1		1.0		CCP	OG2_89419	6	1	0		5		1
PF11_0064	hypothetical protein	85	I	1		1.5			OG2_92898	6	0	0		4		1
PF11_0141	UDP-galactose transporter	39	I			8.0		UAA; TPT; DUF6	OG2_72118	6	4	>10		3		1
PF10_0223	hypothetical protein	49	I			4.3			OG2_92751	6	0	0		3		1
PF11_0344	AMA-1	72	II	1		1.5		AMA-1	OG2_81783	6	3	no		2	19	0.95
PFD0430c	SPECT2 (PLP1)	94	II	1		1.5		MACPF	OG2_81960	6	3	2			26	0.96
**PF14_0074**	**hypothetical protein**	47	II			0.5			OG2_84483	6	1	1			20	1
MAL13P1.212	SPECT1	28	II	1		0.5			OG2_105334	4	0	0			16	0.94
PFL0800c	CelTOS	20	II	1		0.5			OG2_92921	5	0	0			6	1
PFB0570w	SPATR	29	II	1		0.8		TSP1	OG2_89620	6	1	0			5	1
**PFF1195c**	**hypothetical protein**	75	II	1	1	1.3			OG2_89698	6	0	1			4	1
PFE0395c	hypothetical protein (Pf38)	41	II	1	1	1.3		s48_45	OG2_93113	6	0	0			3	1
PFC0905c	hypothetical protein	360	III	1		1.0			OG2_86694	6	0	0	78	133	27	0.99
PF13_0233	myosin a	92	III			0.8		Myosin_head	OG2_70807	6	5	>10	2	39	49	1
**PFD0425w**	**hypothetical protein**	113	III	1		1.8			OG2_86498	6	2	0	3	42	38	1
PF13_0201	TRAP	65	III	1		1.8		VWA; TSP1	OG2_79098	6	3	5	1	6	35	1
PFC0210c	CS	43	III	1	1	1.0	1	TSP1	OG2_87352	1	2	4	2	5	9	1
PFB0325c	cysteine protease	78	III			0.5		Pept_C1	OG2_93012	6	0	0		16	23	1
PFC0180c	membrane skeletal protein	98	III			0.8			OG2_89543	6	1	0		9	25	1
MAL8P1.73	hypothetical protein	141	III			3.3			OG2_84418	6	2	0		16	13	1
**PFA0205w**	**hypothetical protein**	93	III			4.0			OG2_92998	6	0	0		7	22	0.97
PF13_0234	phosphoenol pyruvate carboxykinase	66	III			1.0		PEPCK_ATP	OG2_72511	6	3	>10		8	17	0.93
PF08_0008	hypothetical protein	85	III	1	1	2.0			OG2_89440	6	1	0		3	22	0.93
PFL1165w	hypothetical protein	96	III			0.8	1		OG2_93001	6	0	0		15	6	1
MAL13P1.254	hypothetical protein	31	III	1		2.0			OG2_92954	6	0	0		11	9	1
PF14_0495	hypothetical protein	249	III	1		2.0			OG2_78524	6	3	5		12	6	1
PF14_0286	glutamate dehydrogenase	57	III	1	1	0.5		ELFV_dehydrog N, ELFV_dehydrog	OG2_70937	6	1	>10		3	12	1
**MAL8P1.66**	**hypothetical protein**	26	III	1		1.3	1		OG2_72432	5	3	>10		5	4	1

1 Genes shown in bold been further characterized by functional analysis through targeted gene disruption (see Table 4).

2 Classification of proteins based on their protein expression patterns: group I (ODS-enriched), group II (SGS-enriched), and group III (ODS/SGS-enriched).

3 Abbreviations used for motif predictions: are Signal Peptide (SP), Glycosylphosphatidylinisotol anchored proteins (GPI), Trans Membrane regions (average of number of TM regions predicted by four algorithms) and N-terminal PEXEL (Plasmodium export element within first 200 amino acids obtained from http://www.plasmodb.org). Abbreviations for predicted SMART domains: Apical membrane antigen (AMA), Complement control proteins (CCP), Domain of Unknown Function (DUF), Glu/Leu/Phe/Val dehydrogenase dimerisation domain (ELFV_dehydrog_N), Glutamate/Leucine/Phenylalanine /Valine dehydrogenase (ELFV_dehydrog), Domain containing 5 cysteine conserved residues (GCC2_GCC3), Metal dependent phosphohydrolases with conserved ‘HD’ motif (HDc), Membrane-attack complex /perforin domain family (MACPF), Phosphoenolpyruvate carboxykinase (PEPCK_ATP), Papain family cysteine protease (Pept_C1), Sexual stage antigen s48/45 domain (s48-45), Thrombospondin type 1 (TSP1), Triose-phosphate Transporter family (TPT), UAA transporter family (UAA), Von Willibrand factor type A domain (VWA).

4 Orthology predicition from OrthoMCL database (http://www.orthomcl.org) shows OrthoMCL group, nr species Plasm (the number of *Plasmodium* species, additional nr species Api (number of additional Apicomplexan species without *Plasmodium* species), additional species other (number of species without Apicomplexan species (including *Plasmodium*)).

5 OOC – oocysts; ODS - oocyst-derived sporozoites; SGS - salivary gland sporozoites. The mosquito fraction (mosq fr.) of a protein is calculated by the number of detected peptides in mosquito stages divided by the sum of the number of peptides in mosquito and blood stages.

### Functional characterization of sporozoites-specific proteins

In total eight genes identified in this study were selected (Table 4) for functional analysis by targeted gene disruption of their corresponding orthologs in *P. berghei*, specifically, 3 ODS specific genes (Group I), 2 SGS specific genes (Group II) and 3 from Group III (shared ODS/SGS). The sequences of the eight *P. berghei* gene orthologs (as well as their corresponding up and downstream sequences) were retrieved from the on-line *Plasmodium* genome databases, http://www.plasmodb.org and http://www.genedb.org/genedb/pberghei. However, for 4 of the 8 genes the *P. berghei* orthologs were fragmented and complete genes were manually assembled from a number of different *P. berghei* sequences by performing BLAST sequence searches of the full length *P. falciparum* genes against the *P. berghei* genome and closing gaps by PCR; details of the *P. berghei* orthologs, assemblies and generation of knock-out constructs is available in Figure S3 and Table S5. The generation of mutant parasites was performed in the GFP-expressing reference line of *P. berghei* (i.e. line 507cl1) by standard genetic transfection of constructs for gene-disruption by double cross-over homologous recombination [47]. Genotype analysis of mutant parasites by Southern analysis of genomic DNA and diagnostic PCR was performed using well established methods [48] and details of these analyses are shown in Figure S3.

**Table 4:** Genes selected for further functional analysis of their encoded proteins through targeted disruption of their orthologs in the *P. berghei* rodent model.

*P. fal.* Ac. Nr.	Group (1)	*P. berghei* accession nr.	Exp nr (mutant) (2)	Successful disruption (3)	Oocyst no. mean (s.d.) (2–4 exp) (4)	spor. no. mean (s.d.) (2–3 exp) (5)	spor. infectivity to mice (mosquito bite) (6)	Phenotype (7)	Remarks (8)
Control (WT)		-	-	-	169 (39)	104000 (14500)	4 (n = 4)	wild type	-
PF11_0528	I	PB001073.00.0/PB000529.02.0/PB000863.00.0	800, 836	Yes (2×)	160 (48)	64800 (12500)	2 (n = 2)	Wild type	Redundant function?
PF14_0435	I	PB101363.00.0/PB000829.02.0/PB105739.00.0	802, 838	Yes (2×)	181 (60)	0	0 (n = 4)	Complete block of sporozoite development inside oocyst	
Pf14_0607	I	PB000015.03.0	803, 829, 839	No (3×)	-	-	-	-	Essential for blood stage?
PF14_0074	II	PB000372.00.0	807, 842	Yes (2×)	153 (27)	80200 (16000)	2 (n = 2)	Wild type	Redundant function?
PFF1195c	II	PB107027.00.0/PB107193.00.0/PB001101.03.0	801, 837	Yes (2×)	165 (47)	97300 (12200)	2 (n = 2)	Wild type	Redundant function?
PFD0425w	III	PB000251.01.0	806, 841	Yes (2×)	154 (59)	375 (125)	0 (n = 4)	Arrest of sporozoite egress from oocyst	Oocyst sporozoites are infective to mice
PFA0205w	III	PB301531.00.0	809, 830, 844	No (3×)	-	-	-	-	Essential for blood stage?
MAL8P1.66	III	PB402680.00.0	808, 843	Yes (2×)	188 (51)	2625 (415)	0 (n = 4)	(Partial) block of sporozoite development inside oocyst	Few sporozoites formed; not infective to mice

1 Classification of proteins based on their protein expression patterns. Group I ODS-enriched, group II SGS-enriched, and group III ODS/SGS-enriched.

2 The experiment numbers ( = mutant number) of 2–3 independent transfection experiments.

3 Successful disruption was determined by Southern analysis of separated chromosomes and diagnostic PCR of selected, pyrimethamine resistant parasites as shown in Figure S3 and Table S5.

4 Mean number of oocysts per mosquito and standard deviation (s.d).

5 Mean number of salivary gland sporozoites per mosquito and standard deviation (s.d).

6 Infectivity of sporozoites was tested by infecting mice by mosquito bite. The number of mice that showed blood stage infection comparable to wild type infections is shown.

**Figure 3:**
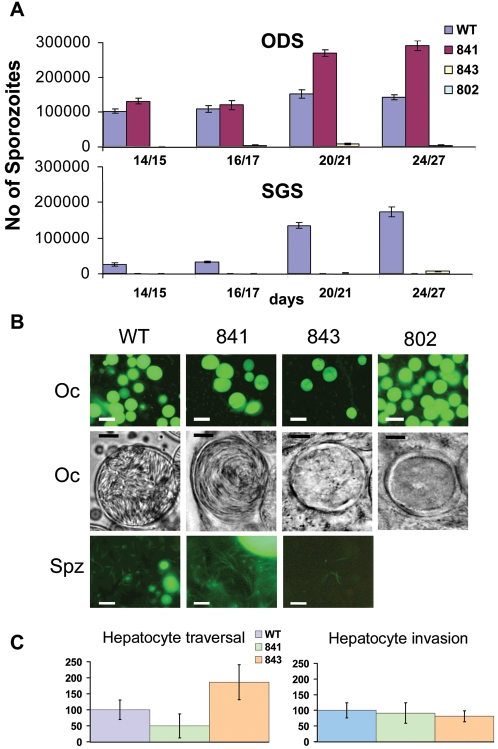
Phenotypic characterization of *P. berghei* mutants (841cl1, 843cl1, 802cl1) with disrupted genes. (A) Numbers of oocyst-derived sporozoites and salivary gland sporozoites per mosquito from day 14 till day 27 post mosquito infection. Scale bars in Figure 3a indicate 50 µm. Wild type (WT) sporozoite numbers are shown in blue bars, 841 clone (PB000251.01.0/PFD0425w) gene disruptant sporozoite numbers in purple, 843 clone (PB402680.00.0/MAL8P1.66) gene disruptant sporozoite numbers in yellow, and 802 clone (PB101363.00.0-PB000829.02.0-PB105739.00.0/PF14_0435) gene disruptant sporozoite numbers are shown in pale blue. (B) Oocysts and sporozoites of the three mutant lines. Upper panel: GFP-expressing mature oocysts at day 10 after infection. Middle panel: Representative images (phase contrast microscopy) of mature (day 12) oocyst. Sporozoite formation in mutant 841 (PB000251.01.0/PFD0425w) is same as WT whereas in lines 843 (PB402680.00.0/MAL8P1.66) and 802 (PB101363.00.0-PB000829.02.0-PB105739.00.0/PF14_0435) sporozoite development is either affected (i.e. 843) or completely absent (i.e. 802). Lower panel: GFP-expressing sporozoites (released by mechanical rupture of oocysts at day 18–20). Scale bars in Figure 3b indicate 12 um. (C) Hepatocyte traversal and invasion of oocyst derived sporozoites (841, PB000251.01.0/PFD0425w)) and salivary gland sporozoites (843, (PB402680.00.0/MAL8P1.66)) compared to WT sporozoites similarly mechanically liberated from oocyst. Bars represent the average percentage of HepG2 cell traversal and invasion relative to wild type. Scale bars in Figure 3c indicate 12 um.

7,8 See Figure 3 for details of the characterization of the 3 mutants with a phenotype different from wild type.

OOC – oocysts; ODS - oocyst-derived sporozoites; SGS - salivary gland sporozoites.

It was not possible to select mutant parasites for two genes, one belonging to Group I (orthologous to PF14_0607) and the other belonging to Group III (orthologous to PFA0205w) in 3 independent transfection experiments, suggesting that both these proteins may have an additional and essential role during blood stage development. For the remaining 6 genes mutants were generated in two independent transfection experiments per gene (Table 4) and correct disruption of the target genes was shown for all mutants (Figure S3). All 6 mutant lines showed normal asexual growth and also gametocyte and ookinete production that was comparable to wild type parasites (data not shown). As an initial phenotype screen of mosquito stage development, uncloned parental populations of the 6 mutant lines were allowed to infect mosquitoes. Oocyst numbers and salivary gland sporozoite numbers were determined at day 6 and 20 after infection, respectively, and infected mosquitoes were allowed to feed at day 20–22 on naïve mice. In 3 out of the 6 mutant lines (orthologous to ΔPF11_0528, ΔPF14_0074 and ΔPFF1195c) parasite development inside the mosquito (oocyst number and salivary gland sporozoites number) was not significantly different from wild type parasites (Table 4). After infection of mice by bite of mosquitoes infected with any of these three mutant lines, all mice developed parasitemias between 0.1 and 0.5 at day 4 after infection, indicating ‘wild type’ infectivity of the sporozoites of these 3 mutants. Genotype characterization by Field Inverse Gel Electrophoresis (FIGE) analysis and diagnostic PCR of blood stage parasites after mosquito transmission of these 3 mutants revealed the correct gene disruption genotype in blood stages of all 3 mutants, demonstrating normal mosquito transmission of the mutant, rather than breakthrough of wild type parasites (Figure S3). The lack of a clear effect of disruption of these 3 genes on sporozoite production and infectivity to the mammalian host suggests the existence of significant redundancy in the function of these mosquito stage specific proteins.

The remaining 3 mutant lines (orthologous to ΔPF14_0435, ΔPFD0425w and ΔMAL8P1.66) showed an aberrant development during mosquito development. The phenotypes of cloned lines of these mutants were therefore analyzed in more detail. Clones of all 3 gene-disrupted lines produced wild type numbers of oocysts ranging from 150–250 oocysts per mosquito on day7/8 post infection. The development of parasites deficient in PB000829.02.0 (orthologue of PF14_0435; line 802cl1) was blocked at the developing oocyst stage and no sporozoite formation was detectable within the oocysts by either fluorescence or phase-contrast microscopy (Figure 3). This early function in sporozoite development of this protein is in agreement with its presence in ODS and absence in SGS. The development of parasites deficient in PB000251.01.0 (orthologue of PFD0425w; line 841cl1) was normal up to the formation of mature oocysts which contain sporozoite numbers similar to wild type oocysts (Figure 3). However, only very few sporozoites were observed in the hemocoel and salivary glands (ranging from 0–625 per mosquito in different experiments (Figure 3)), suggesting that egress of sporozoites from mature oocysts is severely affected. This is also apparent from the accumulation of sporozoites in oocysts from day 20 post infection, where higher levels of oocyst-sporozoites were counted compared to wild type. Furthermore, day 24–27 infected mosquitoes containing mature oocysts with sporozoites were unable to infect mice in standard feeding experiments (2 experiments; 2 mice per experiment). However, when sporozoites were collected from oocysts by liberating them using mechanical rupture and these were used to infect mice by intravenous injection (1–2×10^6^ sporozoites) they were infective to mice comparable to wild type ODS (2 experiments each with 2 mice). Additionally, if such oocyst-extracted sporozoites were used in *in vitro* hepatocyte invasion assays they showed hepatocyte traversal and invasion that was not significantly lower than sporozoites from wild type sporozoites also mechanically extracted from oocysts (Figure 3). The ‘wild type’ infectivity of oocyst-liberated sporozoites to the mammalian host strongly indicates that normal and viable sporozoites are formed within the oocysts and that the absence of protein PB000251.01.0 prevents the release of these sporozoites from the oocyst. Finally, the development of parasites lacking PB402680.00.0 (orthologous to MAL8P1.66; line 843cl1) was largely blocked at the oocyst stage. However, low numbers of sporozoites were formed that were able to invade the salivary gland (2750–6250 oocyst sporozoites per mosquito and 875–6600 SGS per salivary gland). Despite the low numbers of sporozoites that emerge from the oocyst, salivary gland invasion appears not to be affected since ODS and SGS numbers were comparable. In contrast to sporozoites of mutant 841cl1, salivary gland sporozoites of 843cl1 injected either intravenously (1×10^4^ sporozoites) or by mosquito bite were not infective for mice (2 experiments with 2 mice). Interestingly, 843cl1 sporozoites demonstrated the same or greater hepatocyte traversal rate than wild type sporozoites and they were also able to traverse and invade hepatocytes *in vitro* (Figure 3). This suggests that the lack of sporozoite infectivity to mice may be due to a defect in liver stage development after invasion of the hepatocyte.

## Discussion

The proteome analyses of the three mosquito stages of *Plasmodium falciparum*, oocysts, oocyst-derived sporozoites and salivary gland sporozoites, resulted in the identification of 728 proteins of which 250 are ‘mosquito stage specific’, having not been detected in our previous analysis of blood stage parasites [15]. Although the total number of proteins identified in the mosquito stages is lower compared to blood stages [15], which is in all likelihood due to sample purity and not reduced protein expression, we show a clear developmental progression of the parasite through the mosquito that is reflected in changes of its protein repertoire.

Analysis of the ‘stage specificity’ of proteins in six different life cycle (mammalian and mosquito) stage proteomes demonstrated that expression of proteins restricted to a single stage ranges from 12 to 28% with the highest percentage of ‘stage specificity’ in the gametocyte and reaching 24% in ODS. The 478 proteins common to blood and mosquito stages are significantly enriched in house keeping proteins involved in metabolic processes. The absence of specific enrichment of GO annotations in the 250 proteins of the mosquito stage specific proteome can most likely be ascribed to the fact that a relatively small number of these proteins posses a GO designation. Many of the mosquito stage specific proteins are still annotated as hypothetical and probably have functions that are specific for sporozoites and/or *Plasmodium*. This concept is supported by the observation that 15 of the 23 *Plasmodium* proteins known to have a sporozoite specific function are present in the 250 mosquito stage proteins identified in this study, a 4–5 fold enrichment. Moreover, their stage specific expression in our different proteomes also confirms that in general the timing of protein expression coincides with observation of function as inferred from gene deletion studies. For example, proteins involved in the traversal and invasion of the hepatocyte (e.g. SPECT1/2, CelTOS, AMA-1, STARP, TRSP, Pf36p and P36 (Table 1)) are either exclusively or much more highly expressed in SGS than ODS. Such changes in protein composition and abundance demonstrate that sporozoites go through dynamic changes and may exist as clearly defined developmental stages – currently ODS and SGS – that express stage specific proteins. These clear differences seem unexpected in the light of the morphological similarity of the two stages but on the other hand are in good agreement with the significant rise in mammalian host infectivity observed during the maturation and migration of sporozoites from oocysts to salivary glands [20],[39]. These changes are not only restricted to proteins directly involved in these processes, but extend also to enzymes implicated in metabolic housekeeping processes such as glycolysis, production of NADPH and the TCA cycle that might be expected to coincide with subcellular reorganisation at the level of the organelles. Mature, salivary gland sporozoites might be considered to be in the resting phase (G0) of the cell cycle and are able to persist and remain infectious within the salivary glands of the mosquito for the remainder of its life. Therefore, the abundance and storage of these proteins may suggest that the salivary gland sporozoite contains stockpiles of proteins which are deployed only upon activation in the vertebrate host and growth (G1) and multiplication (S, M phases) inside the hepatocyte. Alternatively, some of these proteins could specifically be required by the parasite in the salivary glands of the mosquito host and therefore do not depend on activation in the vertebrate host.

Protein and gene expression studies of SGS have previously been performed in *P. falciparum* [10],[16] as well as for the rodent parasites *P. berghei* [11],[20],[21] and *P. yoelii* [18],[19]. The relatively low overlap between the proteins detected in the various proteomes of sporozoites can in part be ascribed to the difficulties in collecting material of sufficient purity and quantity. This limitation results in the frequent sequencing of peptides derived from mosquito proteins which reduces the total number of identified parasite proteins. However, both the degree of overlap between the proteomes and the degree of certainty in protein calling can be improved if more strict selection criteria are used for protein calling [31]. When we compared only proteins that were identified by at least 2 or more fully-tryptic peptides in all datasets (i.e. ours, Florens [10] *P. falciparum* SGS and Hall [11] *P. berghei* SGS) we found a greater than 50% overlap in proteins. Moreover, in the Hall *P. berghei* SGS and OOC proteomes it is observed that more than 80% of these proteins have a direct ortholog in *P. falciparum*. Further, when we again only compare ‘fully tryptic proteomes’ we find 75% of the *P. berghei* SGS proteins are also expressed in the SGS of *P. falciparum* indicating that sporozoites of different *Plasmodium* species employ similar processes of maturation and invasion. Despite the relatively low overlap in total numbers of proteins detected in the different proteomes, there is good correlation of protein abundance between our SGS proteome and the previously reported SGS proteome of *P. falciparum* [10] based on peptide counting methods.

Interestingly, nearly all the expressed genes of *P. yoelii* sporozoites detected by EST analyses [18] are also present in our proteome. Similarly, in a recent microarray analysis of *P. yoelii*, where 5500 expressed genes were measured in the ODS/SGS stages we find that all of our 601 (i.e. 601 of the 728 *P. falciparum* genes that have a *P. yoelii* ortholog) mosquito stage specific *P. falciparum* proteins are also detected as mRNA [22].

We found a lower percentage of shared proteins between our proteome and the transcripts detected in sporozoites of *P. berghei* [11],[20],[21]. The variation in overlap between the various proteome and transcriptome studies is certainly influenced by the varying and often small number of identified genes/proteins and indicates that a comprehensive expression profile of the salivary gland sporozoite has still to be realized. Comparison of mRNA species detected in *P. falciparum* SGS [22] with our proteome showed that for a large percentage of genes, mRNA production coincides with the presence of its protein (463 mRNA species for 477 proteins; 97%). The simultaneous presence of transcripts and protein expression has also been observed during blood stage development, supporting the ‘just in time’ model [9]. However, more than 2100 genes demonstrate an up-regulation of transcription in sporozoites [22], many of which were not detected as a protein in the various proteome studies. Moreover, a low correlation exists between the abundance levels of our SGS proteins (i.e. by emPAI) and the mRNA abundance of previously reported large-scale SGS transcriptome studies (i.e. r = 0.31–0.33; [16],[22]). This is in line with the observations made by Le Roch et al (2004) where transcript levels are not always well correlated with same stage protein expression, suggesting a delay between mRNA and protein accumulation [49]. It is interesting to speculate whether these differences in expression between RNA and protein could be in part explained by translation repression as is observed in gametocytes that contain pools of translationally repressed transcripts that are only translated following zygote formation [11],[50],[51]. However, as discussed above, the proteome of sporozoites may not be comprehensive enough to draw conclusions about the relationship between specific mRNA and protein expression patterns.

The sporozoite proteomes, despite not being exhaustive, provide for the first time information on parasite protein expression both at the mosquito midgut and salivary gland stages. This has allowed for the identification of hitherto uncharacterized proteins which in turn has informed the selection of genes for targeted orthologous gene disruption studies in the rodent malaria parasite, *P. berghei*. Mutant *P. berghei* parasites lacking mosquito stage specific proteins have proven to be an efficient way to obtain an understanding into the function of such proteins [52]. We were able to generate 6 mutants for 8 hypothetical proteins that were selected from our proteomes for further functional analysis in *P. berghei* of which 3 showed distinct phenotypes, demonstrating an important and essential role of these proteins in sporozoite development and maturation. The knock-out parasite lines of 3 genes that do not exhibit a clear phenotypic difference from wild type parasites indicate either a redundancy in function for the proteins encoded by these genes or else phenotypes that are presently too subtle for us to detect with our current methodologies. However, functional redundancy is a well-established phenomenon for a number of *Plasmodium* proteins that are expressed in the blood and sexual stages of the parasite [53]–[55].

The *P. falciparum* protein PF14_0435 is highly and exclusively expressed in sporozoites obtained from the oocyst stage and the phenotype of the orthologous gene knock-out mutant in *P. berghei*, 802cl1, is an abnormal development of the oocyst and the complete absence of sporozoite production. This example demonstrates not only the validity of the orthologous gene studies in *P. berghei* but also the informative power of this combination of proteome-reverse genetic approach in the characterization of proteins at discreet stages of the parasite life-cycle. Furthermore, the number of oocysts produced by the 802cl1 mutant is not different from wild type levels and a defect appears to occur prior to sporozoite development indicating that the role of PF14_0435 is upstream of sporozoite production. The phenotype of a second *P. berghei* mutant, line 841cl1, which lacks the orthologue of PFD0425w closely resembles the egress defects observed with the cysteine protease ECP1 (or SERA8 in *P. falciparum*) and CS mutants that are mutated in their thrombospondin repeat; where sporozoites are unable to exit from midgut oocysts [44],[56]. Although ECP1 mutant sporozoites are not infectious, it has been suggested that ECP1 may be involved in the cleavage of CS and thereby release of sporozoites from the oocyst [4]. Interestingly, while oocyst-derived sporozoites that lack ECP1 or express mutated CS are not infective to mice, the mechanically liberated oocyst-derived sporozoites of mutants lacking PFD0425w are able to establish an infection in mice by i.v. inoculation, and this implies that PFD0425w - in contrast to ECP1 - has no additional function during infection of the mammalian host. Its role appears to be restricted and directly involved in sporozoite release from the oocyst and has a more immediate/causative function in the release of sporozoites from oocysts. In contrast, the protein MAL8P1.66 appears to have multiple roles during sporozoite development within the oocyst and infectivity to the mammalian host. Mutants lacking this protein (i.e. line 843) are affected in the production of sporozoites within oocysts. However, the low numbers of sporozoites formed are able to invade salivary glands and hepatocytes *in vitro* but are unable to infect mice, suggesting an additional role during further development inside the hepatocyte. Interestingly and in line with the expectation, the expression of MAL8P1.66 has recently been identified in liver stage of *Plasmodium* [57]. The exact role during development of the liver stages awaits further analysis.

This study sheds light not only on the development and maturation of the malaria parasite in an *Anopheles* mosquito but also identifies proteins that are uniquely synthesized as the sporozoite becomes increasingly infectious to humans. Infection initiated by injection of *P. falciparum* sporozoites into humans represents the culmination of many precise, sequential and critical developmental steps of the malaria parasite through the mosquito. Moreover, transmission is a bottle neck in the life cycle of *Plasmodium* and the full maturation of sporozoites is essential in the survival of the parasite. The changes in the different sporozoite proteomes documented here emphasise that each event from oocyst development to egress and invasion of salivary gland and injection is tightly regulated. Intervention studies are now being conducted that aim to exploit the tightly regulated pathways that the parasite has evolved to ensure transmission. This has been recently demonstrated with the use of genetically attenuated sporozoites that have rapidly become an important focus in the development of new vaccines. The disruptions of individual genes that encode sporozoite proteins sufficiently weakens the parasite such that development in the liver is blocked, enabling the mammalian host to generate a strong protective immunity against subsequent infection. Clearly, the targeted disruption of genes encoding proteins identified in this study, which are involved in essential mature sporozoites functions, namely hepatocyte traversal, invasion and intracellular survival may also accelerate the identification of new protective attenuated parasite lines. Understanding the sporozoite and all its various developmental steps during the establishment of an infection continues to represent a promising approach in the hunt for new weapons in the fight against malaria.

## Materials and Methods

### Collection of *P. falciparum* oocysts and sporozoites

*Anopheles stephensi* mosquitoes (Sind-Kasur strain, 3–5 days old) [58] were infected with *P. falciparum* gametocytes (NF54) [59] by membrane feeding. Unfed and partially fed mosquitoes were removed and fully fed mosquitoes were kept at 26±1°C at 80% humidity. After one day, a 5% glucose solution soaked in cotton wool was offered to the mosquitoes and mosquitoes were allowed to take an extra (uninfected) blood meal at day 8–10 after infection [60]. Oocysts and oocyst-derived sporozoites were collected from midguts at 7–8 and 13–14 days after infection, respectively. Approximately 100–200 mosquito midguts were hand-dissected and homogenized in a home made glass tissue grinder in 200 µl of PBS pH 7.2 at 4°C. Salivary gland sporozoites were collected from salivary glands 18–22 days after infection. Approximately 70 salivary glands were hand-dissected and treated in a similar way as the oocyst samples. For the parasite preparations (OOC, ODS and SGS), four, three and two batches respectively were generated and processed further for analysis by nLC-MS/MS.

### Sample preparation for Mass Spectrometry analysis

In order to estimate the number of sporozoites in the samples described above the total number of oocyst and salivary gland sporozoites per mosquito was determined as follows: midguts and salivary glands were dissected from 10 mosquitoes at day 13 and day 22 after feeding respectively. The midguts/salivary glands were homogenized in a home made glass grinder in 1000 µl of PBS pH 7.2 and sporozoites were counted in a Bürker-Türk counting chamber using phase-contrast microscopy (1–1.6×10^5^ sporozoites obtained from salivary glands of one mosquito, and 0.5–5×10^5^ sporozoites per mosquito midgut). Parasites samples from mosquito midguts and salivary glands (approx. 1–4×10^7^ ODS and SGS sporozoites, and 1–2×10^4^ oocysts from 65–200 mosquito midguts) were divided into a soluble and insoluble fraction by a freeze–thawing procedure similar to the parasite sample preparation procedure of blood stages [15]. Complex protein mixtures of both fractions derived from different batches were extracted in SDS–polyacrylamide gel electrophoresis (PAGE) loading buffer and subsequently separated into 10 or 22 fractions per sample batch after electrophoresis on a 10% protein gel to reduce protein complexity, allowing protein identification by 1D LC-MS/MS. Because parasite samples were contaminated with mosquito host proteins (from midguts and salivary glands), we also analysed an increased number of gel slices (22 slices per gel) compared to 10 slices used in our previous analysis for *P. falciparum* blood stages. Gel slices were treated with dithiothreitol and iodoacetamide and digested by trypsin as described before [15].

### Nano–liquid chromatography tandem mass spectrometry

The nLC-MS/MS procedure as described for the analysis of blood stages [15] was used with minor adjustments. Peptide mixtures were loaded onto 100 µm ID columns packed with 3 µm C18 particles (Vydac) and eluted into a quadruple time-of-flight mass spectrometer (QSTAR, Sciex-Applied Biosystems). Fragment ion spectra were recorded using information-dependent acquisition and duty-cycle enhancement. Since the parasite samples were contaminated with host (mosquito) proteins, we measured samples up to four times with exclusion lists to acquire MS/MS spectra of *P. falciparum* peptides. Peptides sequenced in the first run were excluded for sequencing in subsequent runs, peptides from the 2^nd^ run were excluded in the 3^rd^ run etc. This procedure results in an enrichment of low abundant peptides in the second, third and fourth LC-MS/MS run. In total, more than 750 LC-MS/MS runs were acquired resulting in at least 200,000 MS/MS spectra per parasite stage. *Plasmodium* proteins were identified by searching combined protein databases of *P. falciparum* (http://www.plasmodb.org), *Anopheles gambiae* (ftp://ftp.ensembl.org/pub/) and human IPI (ftp://ftp.ebi.ac.uk/pub/databases/IPI/) using the Mascot search algorithm (Matrix Science) with tryptic requirement and 0.2 Da mass tolerance for precursor mass and fragment masses. First ranked peptides (Mascot peptide scores>15) were parsed from Mascot database search html-files with MSQuant (http://www.msquant.sourceforge.net) to generate unique first ranked peptide lists. *Plasmodium* proteins identified by 1–3 three first ranked peptides were verified by manual inspection of the MS/MS spectra in MSQuant or in Mascot. An initial validation filter was applied to the dataset after reversed database searches. A minimal Mascot peptide score of 30 was determined by a reverse database search, which revealed a false positive rate of 17% for proteins identified by 1 peptide with a Mascot peptide score>30, delta score>5), 5% for proteins identified by 2 peptides (average Mascot score>30) and 0.3% for proteins identified by more than 2 peptides. Manual verification for proteins detected by less than 4 peptides substantially decreased the false positive rates and included proteins below this filter. After internal calibration of the peptide masses by MSQuant, an average absolute mass accuracy of 23.5 ppm was obtained for the entire dataset of *P. falciparum* peptides. To remove redundancy on the protein level and to uniquely assign peptides to one protein, the peptides were remapped to PlasmoDB 5.3 annotated genome using the program Protein Coverage Summarizer (http://ncrr.pnl.gov/software/). The collected peptide list of this study (malaria peptides identified in the mosquito stages) is available in Table S1.

### Identified peptide count analysis to determine protein abundance index values

To determine the protein abundance in our samples, mass spectrometric data was analyzed using an identified peptide per protein count analysis to compute the exponentially modified Protein Abundance Index (emPAI) values [16],[22]. EmPAI values for all proteins in Table S1 were calculated as 10^PAI^–1 (PAI = n_observed peptides_/n_observable peptides_). The number of ‘observable’ peptides per protein was calculated from the output of the program Protein Digestion Simulator (http://ncrr.pnl.gov/software/), which computes peptide masses and hydrophobicities of simulated digests of protein databases. Two approaches were chosen to merge data from proteins identified in several slices, runs and batches. The first approach calculates emPAI values per slice for collapsed data of different runs. Per sample batch, emPAI values were subsequently summed over all slices. In cases for 22 gel slices per lane, data of two slices were merged to create a similar number of emPAI fractions for all samples. The second approach calculates emPAI values for merged data of all slices of all runs per sample batch. Both approaches resulted in protein emPAI values in 4 OOC batches (1–2×10^4^ oocysts), 3 ODS batches (1.4–3.8×10^7^ sporozoites) and 2 SGS batches (1.3–2.5×10^7^ sporozoites).

Normalization between different batches was performed according to the median and 20 percent trimmed mean method [61]. Normalization methods and approaches for merging emPAI data were evaluated on performance in correlation studies with mRNA data of *P.falciparum* salivary gland sporozoites [16],[22] (Table S3). Mean protein emPAI values of merged and median normalized data were calculated per stage and have been included in Table S1. This approach was also applied to our proteomic data set of blood stages [15] to calculate normalized emPAI values.

### Correlation between protein expression data from different studies

Values for the level (abundance) of protein expression from different datasets were obtained for all individual proteins by calculated emPAI values. EmPAI values and mRNA levels of microarray analyses were log2 transformed before regression analysis to obtain normal distributions. Pearson correlation between datasets was performed using R (http://www.r-project.org/).

### Gene ontology annotation

Gene Ontology SLIM terms were assigned using “Generic GO (http://go.princeton.edu/cgi-bin/GOTermMapper). A GO enrichment analysis for ‘Biological Process’, ‘Cellular Component’ and ‘Molecular Function’ using default GO association files was performed with “GO Term Finder” (http://go.princeton.edu/cgi-bin/GOTermFinder) where statistical significance (p-value) is calculated based on hypergeometric distribution with Bonferroni multiple testing correction and false discovery rate calculation as described [62]. To perform a GO enrichment analysis with adjusted GO association files, Ontologizer (http://www.charite.de/ch/medgen/ontologizer/) was used where statistical significance (p-value) is calculated as in ‘GO Term Finder’(see above and [63]).

### Selection criteria for ODS, SGS and ODS/SGS proteins

Proteins with more than 90 percent of the peptides detected in the mosquito stages (mosquito fraction>0.9) were divided into three groups (OOC, ODS and ODS/SGS) based on their expression patterns. The mosquito fraction equals n_mosq_/(n_mosq_+n_blood_) where n is the number of unique peptides per protein at the mosquito and blood stages, respectively. The mosquito enriched proteins were further subdivided into 112 ODS-specific proteins expressed in the ODS stage and not SGS (Group I); 74 SGS-specific proteins not expressed in ODS (Group II); and finally 59 Group III proteins that are shared between several mosquito life cycle stages. A further refinement of these groups was based on the following criteria. Only proteins with more than two peptides detected in the mosquito stages (n_mosq_≥3) were considered. In addition, only proteins were selected that contained Signal peptide (SP), Transmembrane (TM) or Glycosylphosphatidylinositol (GPI) domains and combinations of these motifs. Sequence–based prediction data for these domains was retrieved from PlasmoDB (http://www.plasmodb.org) for SP and TM domain predictions based on TMHMM, TMAP, TMHMM2 and TOPRED2 algorithms; from http://gpi.unibe.ch/ for GPI predictions by a Kohonen Self Organizing Map; and from http://smart.embl-heidelberg.de/ for SMART protein domain searches. The number of TM domains is the average of four values obtained from the different TM prediction algorithms. Different criterions were set for combinations of predicted motifs. For less abundant proteins without predicted signal peptide (SP = 0, and 3≤n_mosq_≤15), only proteins with at least 4 predicted TM regions were included (average TM>4). For abundant proteins without signal peptide (SP = 0, n_mosq_>15) proteins with at least 0.5 predicted TM regions (average TM≥0.5) were included. For proteins with predicted signal peptide (SP = 1), all proteins with at least 0.5 predicted TM regions (average TM≥0.5) were included. Finally, all proteins with a predicted GPI anchor (GPI = 1) were selected independent of the presence of predicted signal peptide or TM regions.

### Generation and characterization of gene knockout *P. berghei* parasite mutants

Eight *P. falciparum* proteins were selected for functional analysis by targeted gene disruption of their corresponding orthologs in *P. berghei*. The sequences of the eight *P. berghei* gene orthologs (as well their corresponding up and downstream sequences) were retrieved from the on-line *Plasmodium* genome databases, http://www.plasmodb.org and http://www.genedb.genedb/genedb/pberghei .For *P. berghei* genes with incomplete sequence information in the database (4 out of 8), the complete genes were manually assembled from a number of different *P. berghei* sequences by performing BLAST sequence searches of the full length *P. falciparum* genes against the *P. berghei* genome and closing gaps by PCR and DNA sequencing (see for details Figure S3 and Table S5). Standard plasmid vectors were designed for targeted gene disruption by double cross-over homologous recombination [48]. To replace the protein coding sequences of the target genes with the *dhfr/ts* pyrimethamine resistance marker from *Toxoplasma gondii*, we cloned the 5′ and 3′ flanking regions of the gene of interest up- and downstream of the selection cassette of pl0001; also in MR4 (http://www.mr4.org/). Briefly described for one candidate gene, to generate a PB000829.02.0/PF14_0435 disruption vector, an upstream region (position 74–436 on singleton berg-2274h02.p1k) and a downstream region (position 516–1016 on contig PB_RP2658) - the latter containing the P. berghei orthologous gene PB000829.02.0 - were amplified from *P. berghei* genomic DNA using primer-pairs 2666–2653 and 2654–2655, respectively. The PCR products were digested with *Asp*718 and *Hind*III, or *EcoR*I and *Not*I, respectively, and ligated into plasmid pl0001 yielding targeting plasmid pL1175 (see for further details of all plasmids and the sequence of the primers, Figure S3 and Table S5). All plasmids generated were sequence analysed. Transfection of GFP-expressing ‘wild type’ parasites from the *P. berghei* reference line 507cl1 [64] with linearised targeting constructs, selection and cloning of the mutant parasites were performed according to procedures previously described [48]. Genotypic analysis of transfected parasites was performed by Southern analysis of FIGE separated chromosomes and diagnostic PCR on genomic DNA (details of the primers used for PCR are shown in Table S5).

Phenotype analysis of mutant parasites during blood stage development, quantification of gametocyte production and ookinete development *in vitro* was performed using standard methods as previously described [14],[65]. Mosquito stage development was analysed in *A. stephensi* mosquitoes using standard methods of infection of mosquitoes and analysis of oocyst and sporozoite production and analysis of sporozoite infectivity to C57Bl6 mice [66]. The number of sporozoites in oocysts of mosquito midguts and in salivary glands derived from 10 mosquitoes was determined in quadruplicate as described above for counting *P. falciparum* sporozoites and represented as mean number with standard deviation per stage per mosquito. The capacity of the mutant parasites to infect mice by mosquito interrupted feeding was determined by exposure of female C57Bl6 mice (n = 2–4) to 40–50 mosquitoes, at day 20 after the infectious blood meal. Infection was monitored by analysis of blood stage infection in Giemsa stained films of tail blood at day 4 till day 8 after infection. Infectivity was recorded as ‘wild type’ if mice developed a parasitemia of 0.1–0.5% at day 4 after infection. Infectivity of sporozoites to mice of 2 mutant lines was also determined by intravenous injection of sporozoites that were mechanically liberated by a glass grinder from either midgut oocysts (1–2×10^6^ oocyst sporozoites collected at day 20 from mutant line 841 and wild type line 507cl1) or collected from salivary glands (10^4^ salivary gland sporozoites at day 27 for mutant line 843 and wild type line 507cl1). For obtaining oocysts and salivary gland sporozoites, mosquito midguts or salivary glands were dissected in a drop of RPMI culture medium and the transferred by a custom made needle into a glass grinder after which sporozoites were released by gently grinding. Blood stage infection in mice injected with sporozoites in 200 µl RPMI buffer was monitored as described for infection of mice via mosquito interrupted feeding.

*In vitro* hepatocyte traversal and invasion experiments were performed as described elsewhere [67],[68] by adding purified sporozoites (5×10^4^) to confluent monolayers of HepG2 cells in DMEM medium (note: medium had 10% FCS and 1% PenStrep). Mutant sporozoites were obtained as described above from either oocysts (day 20) or from salivary glands (day 27). Quantification of cell traversal and invasion was accomplished by using a cell-impermeable fluorescent marker molecule, rhodamine-dextran at 1 mg/ml that will visualize parasitized wounded cells specifically but not uninfected HepG2 cells. Sporozoites were incubated with HepG2 cells in the presence of fluorescent dextran for 2 hr, followed by washing the cells to remove the marker and incubation for an additional 24 hours to determine the development of exoerythrocytic forms (EEFs) of the parasite. Hepatocyte invasion was determined by counting the percentage of sporozoites inside dextran-negative cells because parasites do not develop successfully in wounded dextran-positive cells [68]. After fixation of the HepG2 cells, infection was quantified by staining EEFs with monoclonal antibody 2E6 against HSP70 [69] and compared to infection of wild type sporozoites. Hepatocyte cell traversal was determined by counting the percentage of dextran-positive cells 2 hours after adding sporozoites to HepG2 cells, and compared to wild type sporozoite cell traversal. In this procedure, monoclonal antibody 3D11 against CS was used.

### Accession Numbers

All datasets will become available through the official Web site of the Plasmodium genome project, PlasmoDB (http://www.plasmodb.org [70],[71]). In the text and tables most genes and gene products are accompanied with their PlasmoDB Accession Number.

The PlasmoDB accession numbers for other genes and gene products discussed in this paper are for *P. falciparum*: CS (PFC0210c), TRAP (PF13_0201), UIS3 (PF13_0012), P36 (PFD0210c), P36p (PFD0215c), myosin A (PF13_0233), MTIP (PFL2225w), actin (PFL2215w) and F-1,6-BP aldolase (PF14_0425), AMA-1 (PF11_0344), TRSP (PFA0200w), RESA8 (PFB0325c), SPECT1 (MAL13P1.212), SPECT2 (PFD0430c), CelTOS (PFL0800c), STARP (PF07_0006); and for *P. berghei*: UIS4 (PB100551.00.0), ECP1 (PB000649.01.0).

The sequences of the eight *P. berghei* gene orthologs (as well their corresponding up and downstream sequences) that have been analysed in gene-disruption studies were retrieved from the PlasmoDB database (http://www.plasmodb.org) and from the GeneDB database (http://www.genedb.genedb/genedb/pberghei). For 4 out of 8 *P. berghei* genes with incomplete sequence information in the database, the complete genes were manually assembled from a number of different *P. berghei* sequences by performing BLAST sequence searches, PCR and DNA sequencing (see for details Figure S3 and Table S5). Primer sequences used in contig gap closure and location of primers relating to contigs and reads of the revised *P. berghei* gene models have been submitted to GenBank and are provided in Figure S3 and Table S5.

## Supporting Information

Figure S1 Gene Ontology (GO) annotation for proteins from proteomes from two mosquito stages of *P. falciparum*, oocyst-derived sporozoites and salivary gland sporozoites.
(0.05 MB DOC)

Figure S2 Pathway profiling with the number of unique peptides/protein detected in 5 different life-cycle stages (data obtained from this study and from Lasonder et al. ([15]).
(0.09 MB DOC)

Figure S3 Generation and genotype analysis of *P. berghei* mutants with disrupted genes that encode orthologs of mosquito stage proteins of *P. falciparum*.
(5.77 MB PPT)

Table S1 Peptides and proteins of *P. falciparum* identified in proteomes of oocysts, oocyst-derived sporozoites and salivary gland sporozoites. Page ‘Peptides’: peptides identified by nLC-MS/MS. Information provided in the table: 1) life cycle stage, 2) peptide sequence, 3) MCR (mass of charge of parent ion), 4) charge of parent ion, 5) measured mass of peptide, 6) calibrated mass of peptide after internal mass calibration, 7) peptide rank in Mascot searches, 8) peptide score in Mascot searches, 9) Mascot peptide delta score (which equals the Mascot score difference between a rank1 and a rank2 peptide), 10) accession number of protein identification from PlasmoDB version 5.3, 11) protein name, 12 reannotation PlasmoDB 2008)07)15 (genes with the most recently modified annotation by PlasmoDB), 13) protein mass (molecular weight), 14) protein pI (iso-electric point), 15) nr pept)prot)sample (the number of unique peptides per protein), 16) Residue Start (residue nr in the protein sequence of the N-terminal amino acid), 17) Residue End (residue nr in the protein sequence of the C-terminal amino acid), 18) sequence coverage (percentage of the protein covered by the identified peptides), and 19) protein emPAI value. Page ‘Proteins’: The corresponding proteins identified by the sequenced peptides, listing: 1) accession number (PlasmoDB version 5.3), 2) protein name, 3 reannotation PlasmoDB 2008)07)15 (genes with the most recently modified annotation by PlasmoDB) 4) protein mass (molecular weight), 5) protein pI (iso-electric point), 6) number of unique identified peptides, 7) protein emPAI value, and 8) mosquito fraction (which is calculated by the number of detected peptides in mosquito stages divided by the sum of the number of peptides in mosquito and blood stages). Page ‘OOC’: proteins detected in OOC; column headings the same as for Page ‘Proteins’. Page ‘ODS’: proteins detected in ODS; column headings the same as for Page ‘Proteins’. Page ‘SGS’: proteins detected in SGS; column headings the same as for Page ‘Proteins’. Page ‘Mosquito stage specific’: proteins exclusively detected in mosquito stages (mosquito fraction = 1); column headings the same as for Page ‘Proteins’. Page ‘Shared with RBC stages’: proteins detected in mosquito stages and blood stages (0<mosquito fraction<1); column headings the same as for Page ‘Proteins’. Page ‘OOC-enriched’: proteins that are ‘highly enriched’ in OOC (mosquito fraction>0.9); column headings the same as for Page ‘Proteins’. Page ‘ODS-enriched’: proteins that are ‘highly enriched’ in ODS (mosquito fraction>0.9); column headings the same as for Page ‘Proteins’. Page ‘SGS-enriched’: proteins that are ‘highly enriched’ in SGS (mosquito fraction>0.9); column headings the same as for Page ‘Proteins’. Page ‘tRNA ligase’: overview of all tRNA proteins detected in mosquito stages, column headings the same as for Page ‘Proteins’.
(3.44 MB ZIP)

Table S2 Comparison of proteins identified in the mosquito stage proteomes of this study with the proteomes of salivary gland sporozoites (SGS) of *P. falciparum* as reported by Florens and colleagues [10] and the proteomes of oocysts (OOC) and SGS of *P. berghei* as reported by Hall and coworkers [11]. The tables contain information about proteins shared by our analysis and others, where our expression data is presented by the number of unique tryptic peptides per protein. The other data sets are presented in a similar way, but a distinction is made in expression data for detection either by all peptides (tryptic, half (non) tryptic), or by tryptic peptides.
(1.58 MB ZIP)

Table S3 Correlation of protein abundance (emPAI approach) identified in the SGS stage proteome of this study with mRNA levels of the SGS transcriptomes of *P. falciparum* as reported by Le Roch and coworkers [16] and Zhou and coworkers [22]. Tables show Pearson correlation coefficients (r), probabilities (p) and number of shared genes/proteins (n).
(0.02 MB XLS)

Table S4 Comparison of proteins identified in the mosquito stage proteomes with genes transcribed in sporozoites in *P. berghei* and *P. yoelii* as identified by either subtractive hybridization (SSH) or cDNA quantification methods (SAGE). *S-genes:* 25 sporozoite (S) genes identified in a *P.yoelii* SSH screen [18]. *UIS-genes:* 30 UIS genes (Upregulated In Sporozoites) identified in a *P. berghei* SSH screen [20]. *SIS genes:* 123 SIS genes (Sporozoite expressed gene Identified by SAGE (SIS) genes) identified in a *P. berghei* SAGE analysis [21].
(0.06 MB XLS)

Table S5 Primer sequences used in this study. Primers used in KO targeting plasmid construction. Primers used to check for plasmid integration in mutant (KO) parasites. Primers used in contig gap closure and wild type (WT) PCR analysis.
(0.03 MB XLS)
