# Supplementary material for: Proteomic Profiling of Plasmodium Sporozoite Maturation Identifies New Proteins Essential for Parasite Development and Infectivity
Source: PLoS Pathog. 2008 Oct 31;4(10):e1000195. doi: 10.1371/journal.ppat.1000195 (PMC2570797; doi:10.1371/journal.ppat.1000195)
Supplement: Figure S1 — Gene Ontology (GO) annotation for proteins from proteomes from two mosquito stages of P. falciparum, oocyst-derived sporozoites and salivary gland sporozoites. (0.05 MB DOC) [file ppat.1000195.s001.doc]

**Figure S1:**

**Gene Ontology (GO) annotation for proteins from proteomes from two mosquito stages of P. falciparum, oocyst derived sporozoites and salivary gland sporozoites**

**A:**

**GO annotations (class ‘Molecular Function) that are significantly enriched (p < 0.002)**

**for proteins that were detected both in the mosquito stages and in blood stages**

**B:**

**GO annotations (class ‘Cellular Component) that are significantly enriched (p < 0.009)**

**for proteins that were detected both in the mosquito stages and in blood stages**

**C:**

**GO annotations (class ‘Biological Process’) that are significantly enriched (p < 0.008) for proteins that were detected both in the mosquito stages and in blood stages**
