# Supplementary material for: Proteomic Profiling of Plasmodium Sporozoite Maturation Identifies New Proteins Essential for Parasite Development and Infectivity
Source: PLoS Pathog. 2008 Oct 31;4(10):e1000195. doi: 10.1371/journal.ppat.1000195 (PMC2570797; doi:10.1371/journal.ppat.1000195)
Supplement: Figure S2 — Pathway profiling with the number of unique peptides/protein detected in 5 different life-cycle stages (data obtained from this study and from Lasonder et al. ([15]). (0.09 MB DOC) [file ppat.1000195.s002.doc]

**Figure S2: Number of unique peptides/protein and normalized emPAI values detected and determined in 5 different life-cycle stages (data obtained from this study and from Lasonder et al. (2002) Nature 419: 537-542).**

**A**: Proteins associated with the glycolysis pathway for ATP production from 5 *P. falciparum* life cycle stages. ODS (oocyst-derived sporozoites) SGS (salivary gland sporozoites), ASEX (asexual stages trophozoites and schizonts), GCT (gametocytes) and GAM (gametes)

|  |  | **ODS**  pept (emPAI) | **SGS**  pept (emPAI) |
| --- | --- | --- | --- |

| PFF1155w | hexokinase | 0 (0) | 12 (0.74) |
| --- | --- | --- | --- |
| PF14_0341 | glucose-6-phosphate isomerase | 0 (0) | 9 (0.44) |
| PFI0755c | 6-phosphofructokinase, putative | 0 (0) | 0 (0) |
| PF14_0425 | fructose-bisphosphate aldolase# | 19 (9.03) | 21 (13.54) |
| PF14_0598 | glyceraldehyde-3-phosphate dehydrogenase | 9 (2.02) | 24 (13.06) |
| PFI1105w | phosphoglycerate kinase | 2 (0.81) | 15 (1.69) |
| PF11_0208 | phosphoglycerate mutase, putative | 0 (0) | 20 (18.60) |
| PF10_0155 | enolase | 18 (6.94) | 20 (6.23) |
| PFF1300w | pyruvate kinase, putative | 0 (0) | 19 (3.02) |
|  |  | **----** | **----** |

|  | **nr of peptides** | 48 | 140 |
| --- | --- | --- | --- |
|  | **nr of proteins** | 4 | 8 |

**B**: Proteins associated the Pentose phosphate cycle for NADPH production from 5 *P. falciparum* life cycle stages. ODS (oocyst-derived sporozoites) SGS (salivary gland sporozoites), ASEX (asexual stages trophozoites and schizonts), GCT (gametocytes) and GAM (gametes)

|  |  | **ODS**  pept/emPAI | **SGS**  pept/emPAI |
| --- | --- | --- | --- |
| PFF0530w | transketolase, putative | 0 (0) | 4 (0.40) |
| PF14_0511 | glucose-6-phosphate dehydrogenase-6-phosphogluconolactonase | 0 (0) | 8 (0.25) |
| PF14_0520 | 6-phosphogluconate dehydrogenase, decarboxylating, putative | 0 (0) | 9 (0.70) |
| PFL0960w | D-ribulose-5-phosphate 3-epimerase, putative | 0 (0) | 1 (0.39) |
| PF13_0143 | phosphoribosylpyrophosphate synthetase | 0 (0) | 0 (0) |
| PF10_0122 | phosphoglucomutase, putative | 2 (0.19) | 4 (0.40) |
|  |  | **---** | **---** |
|  | **nr of peptides** | 2 | 26 |
|  | **nr of proteins** | 1 | 5 |

**C**: Proteins associated with the carboxylic acid cycle in 5 *P. falciparum* life cycle stages. ODS (oocyst-derived sporozoites) SGS (salivary gland sporozoites), ASEX (asexual stages trophozoites and schizonts), GCT (gametocytes) and GAM (gametes)

|  |  | **ODS**  pept (emPAI) | **SGS**  pept (emPAI) |
| --- | --- | --- | --- |
| PFF0895w | malate dehydrogenase, putative | 0 (0) | 16 (7,15) |
| PFI1340w | fumarate hydratase, putative | 0 (0) | 7 (0,40) |
| PF10_0334 | flavoprotein subunit of succinate dehydrogenase | 0 (0) | 1 (0.07) |
| PF11_0097 | succinyl-CoA synthetase alpha subunit, putative | 0 (0) | 6 (1,25) |
| PF13_0121 | dihydrolipoamide succinyltransferase, putative | 3 (0.45) | 2 (0.40) |
| PF08_0045 | 2-oxoglutarate dehydrogenase e1 component | 3 (0.21) | 4 (0.15) |
| PF13_0070 | branched-chain alpha keto-acid dehydrogenase, putative | 14 (1.96) | 10 (0.89) |
| PF13_0242 | isocitrate dehydrogenase (NADP), mitochondrial precursor | 6 (0.68) | 6 (0.35) |
| PF13_0229 | IRP-like protein | 0 (0) | 25 (1.20) |
| PF10_0218 | citrate synthase, mitochondrial precursor, putative | 0 (0) | 8 (0.53) |
|  |  | **---** | **---** |
|  | **nr of peptides** | 26 | 85 |
|  | **nr of proteins** | 4 | 10 |
