# Supplementary material for: Proteomic Profiling of Plasmodium Sporozoite Maturation Identifies New Proteins Essential for Parasite Development and Infectivity
Source: PLoS Pathog. 2008 Oct 31;4(10):e1000195. doi: 10.1371/journal.ppat.1000195 (PMC2570797; doi:10.1371/journal.ppat.1000195)
Supplement: Figure S3 — Generation and genotype analysis of P. berghei mutants with disrupted genes that encode orthologs of mosquito stage proteins of P. falciparum. (5.77 MB PPT) [file ppat.1000195.s003.ppt]

## Slide 1
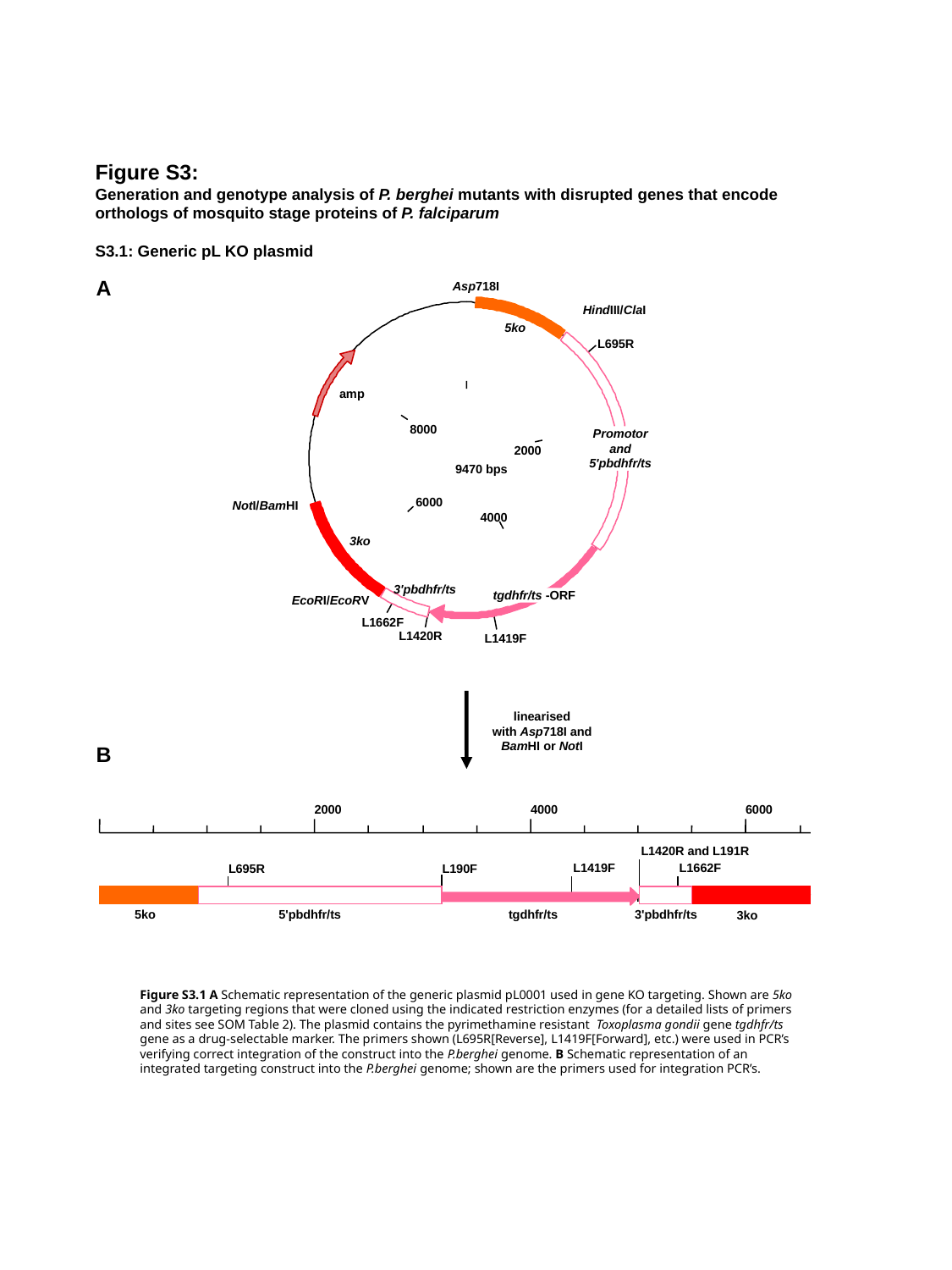

Figure S3:
Generation and genotype analysis of P. berghei mutants with disrupted genes that encode
orthologs of mosquito stage proteins of P. falciparum
S3.1: Generic pL KO plasmid
A
Asp718I
HindIII/ClaI
5ko
L695R
amp
8000
Promotor
and
5'pbdhfr/ts
2000
9470 bps
NotI/BamHI
6000
4000
3ko
3'pbdhfr/ts
EcoRI/EcoRV
tgdhfr/ts -ORF
L1662F
L1420R
L1419F
linearised
with Asp718I and
BamHI or NotI
B
2000
4000
6000
L1420R and L191R
L1419F
L1662F
L695R
L190F
5ko
5'pbdhfr/ts
tgdhfr/ts
3'pbdhfr/ts
3ko
Figure S3.1 A Schematic representation of the generic plasmid pL0001 used in gene KO targeting. Shown are 5ko and 3ko targeting regions that were cloned using the indicated restriction enzymes (for a detailed lists of primers and sites see SOM Table 2). The plasmid contains the pyrimethamine resistant Toxoplasma gondii gene tgdhfr/ts gene as a drug-selectable marker. The primers shown (L695R[Reverse], L1419F[Forward], etc.) were used in PCR’s verifying correct integration of the construct into the P.berghei genome. B Schematic representation of an integrated targeting construct into the P.berghei genome; shown are the primers used for integration PCR’s.

## Slide 2
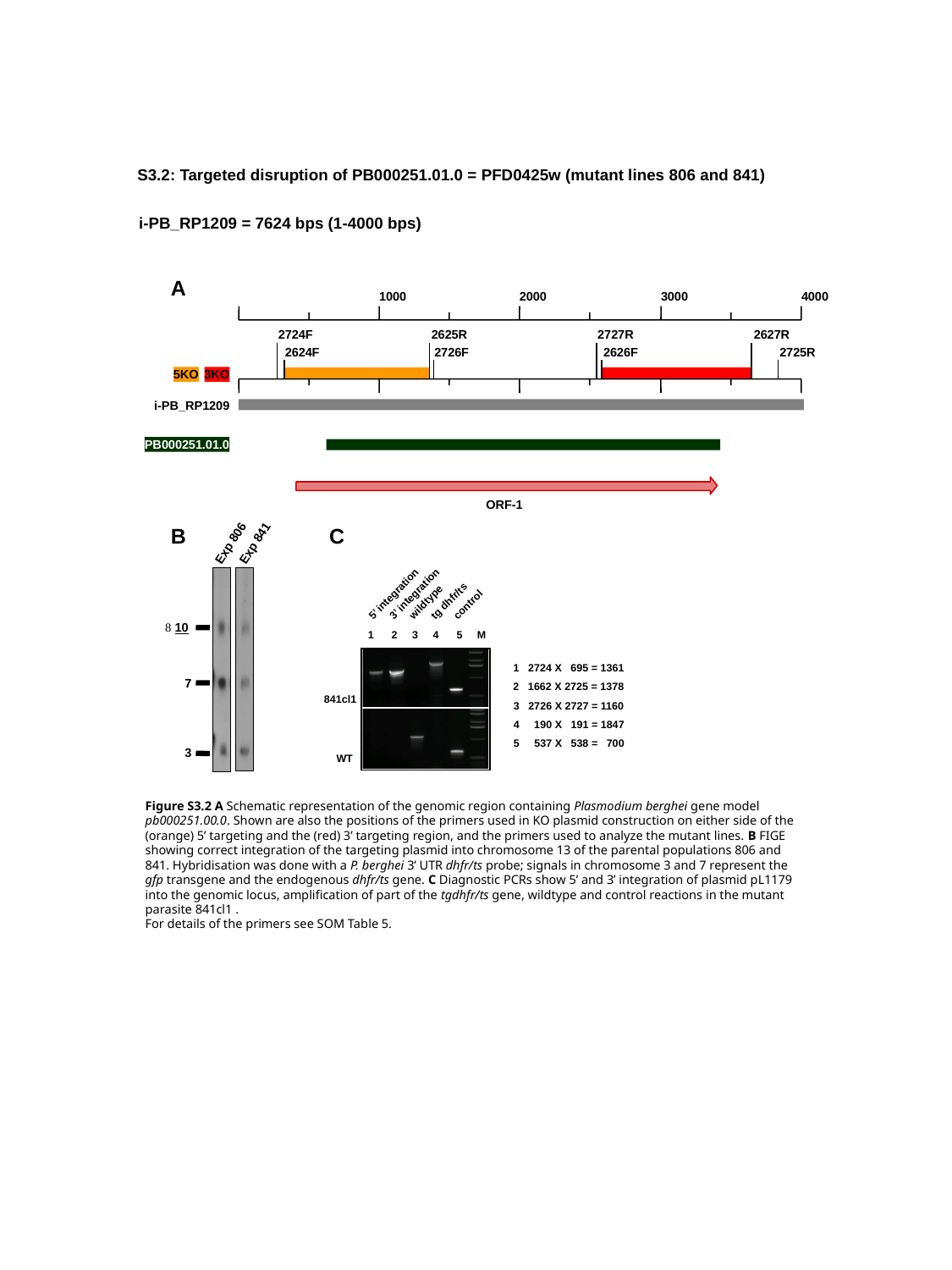

S3.2: Targeted disruption of PB000251.01.0 = PFD0425w (mutant lines 806 and 841)
i-PB_RP1209 = 7624 bps (1-4000 bps)
A
1000
2000
3000
4000
2724F
2625R
2727R
2627R
2624F
2726F
2626F
2725R
5KO
3KO
i-PB_RP1209
PB000251.01.0
ORF-1
Exp 806
Exp 841
 10
7
3
B
C
5’ integration
3’ integration
tg dhfr/ts
wildtype
control
1 2 3 4 5 M
1 2724 X 695 = 1361
2 1662 X 2725 = 1378
841cl1
3 2726 X 2727 = 1160
4 190 X 191 = 1847
5 537 X 538 = 700
WT
Figure S3.2 A Schematic representation of the genomic region containing Plasmodium berghei gene model pb000251.00.0. Shown are also the positions of the primers used in KO plasmid construction on either side of the (orange) 5’ targeting and the (red) 3’ targeting region, and the primers used to analyze the mutant lines. B FIGE showing correct integration of the targeting plasmid into chromosome 13 of the parental populations 806 and 841. Hybridisation was done with a P. berghei 3’ UTR dhfr/ts probe; signals in chromosome 3 and 7 represent the gfp transgene and the endogenous dhfr/ts gene. C Diagnostic PCRs show 5’ and 3’ integration of plasmid pL1179 into the genomic locus, amplification of part of the tgdhfr/ts gene, wildtype and control reactions in the mutant parasite 841cl1 .
For details of the primers see SOM Table 5.

## Slide 3
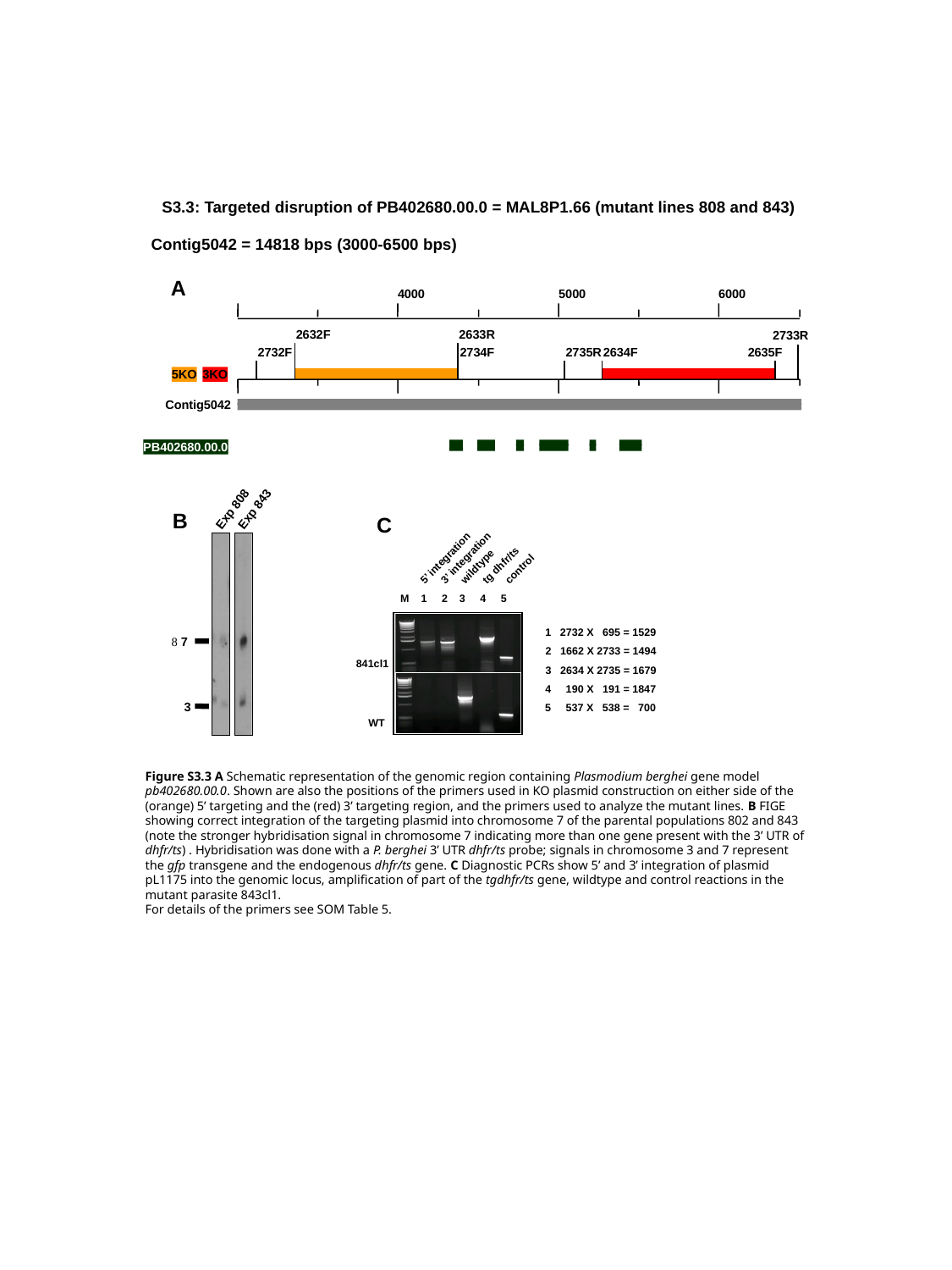

S3.3: Targeted disruption of PB402680.00.0 = MAL8P1.66 (mutant lines 808 and 843)
Contig5042 = 14818 bps (3000-6500 bps)
A
4000
5000
6000
2632F
2633R
2733R
2732F
2734F
2735R
2634F
2635F
5KO
3KO
Contig5042
PB402680.00.0
Exp 808
Exp 843
 7
3
B
C
3’ integration
5’ integration
tg dhfr/ts
wildtype
control
M 1 2 3 4 5
1 2732 X 695 = 1529
2 1662 X 2733 = 1494
841cl1
3 2634 X 2735 = 1679
4 190 X 191 = 1847
5 537 X 538 = 700
WT
Figure S3.3 A Schematic representation of the genomic region containing Plasmodium berghei gene model pb402680.00.0. Shown are also the positions of the primers used in KO plasmid construction on either side of the (orange) 5’ targeting and the (red) 3’ targeting region, and the primers used to analyze the mutant lines. B FIGE showing correct integration of the targeting plasmid into chromosome 7 of the parental populations 802 and 843 (note the stronger hybridisation signal in chromosome 7 indicating more than one gene present with the 3’ UTR of dhfr/ts) . Hybridisation was done with a P. berghei 3’ UTR dhfr/ts probe; signals in chromosome 3 and 7 represent the gfp transgene and the endogenous dhfr/ts gene. C Diagnostic PCRs show 5’ and 3’ integration of plasmid pL1175 into the genomic locus, amplification of part of the tgdhfr/ts gene, wildtype and control reactions in the mutant parasite 843cl1.
For details of the primers see SOM Table 5.

## Slide 4
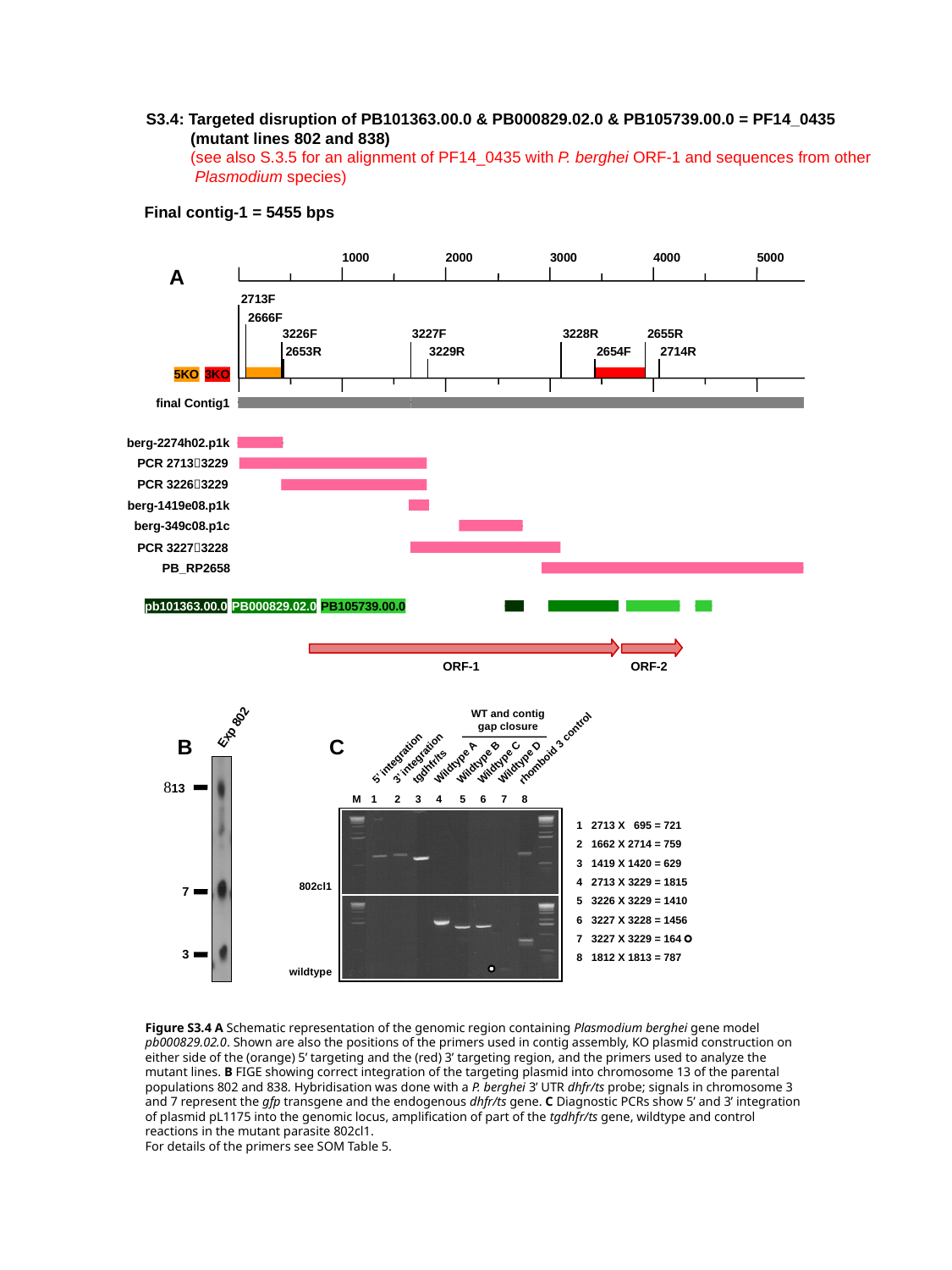

S3.4: Targeted disruption of PB101363.00.0 & PB000829.02.0 & PB105739.00.0 = PF14_0435
 (mutant lines 802 and 838)
 (see also S.3.5 for an alignment of PF14_0435 with P. berghei ORF-1 and sequences from other
 Plasmodium species)
Final contig-1 = 5455 bps
1000
2000
3000
4000
5000
A
2713F
2666F
3226F
3227F
3228R
2655R
2653R
3229R
2654F
2714R
5KO
3KO
final Contig1
berg-2274h02.p1k
PCR 27133229
PCR 32263229
berg-1419e08.p1k
berg-349c08.p1c
PCR 32273228
PB_RP2658
pb101363.00.0
PB000829.02.0
PB105739.00.0
ORF-1
ORF-2
Exp 802
13
7
3
WT and contig
gap closure
rhomboid 3 control
5’ integration
3’ integration
Wildtype A
Wildtype B
Wildtype C
Wildtype D
tgdhfr/ts
M
1 2 3 4 5 6 7 8
1 2713 X 695 = 721
2 1662 X 2714 = 759
3 1419 X 1420 = 629
4 2713 X 3229 = 1815
802cl1
5 3226 X 3229 = 1410
6 3227 X 3228 = 1456
7 3227 X 3229 = 164
8 1812 X 1813 = 787
wildtype
B
C
Figure S3.4 A Schematic representation of the genomic region containing Plasmodium berghei gene model pb000829.02.0. Shown are also the positions of the primers used in contig assembly, KO plasmid construction on either side of the (orange) 5’ targeting and the (red) 3’ targeting region, and the primers used to analyze the mutant lines. B FIGE showing correct integration of the targeting plasmid into chromosome 13 of the parental populations 802 and 838. Hybridisation was done with a P. berghei 3’ UTR dhfr/ts probe; signals in chromosome 3 and 7 represent the gfp transgene and the endogenous dhfr/ts gene. C Diagnostic PCRs show 5’ and 3’ integration of plasmid pL1175 into the genomic locus, amplification of part of the tgdhfr/ts gene, wildtype and control reactions in the mutant parasite 802cl1.
For details of the primers see SOM Table 5.

## Slide 5
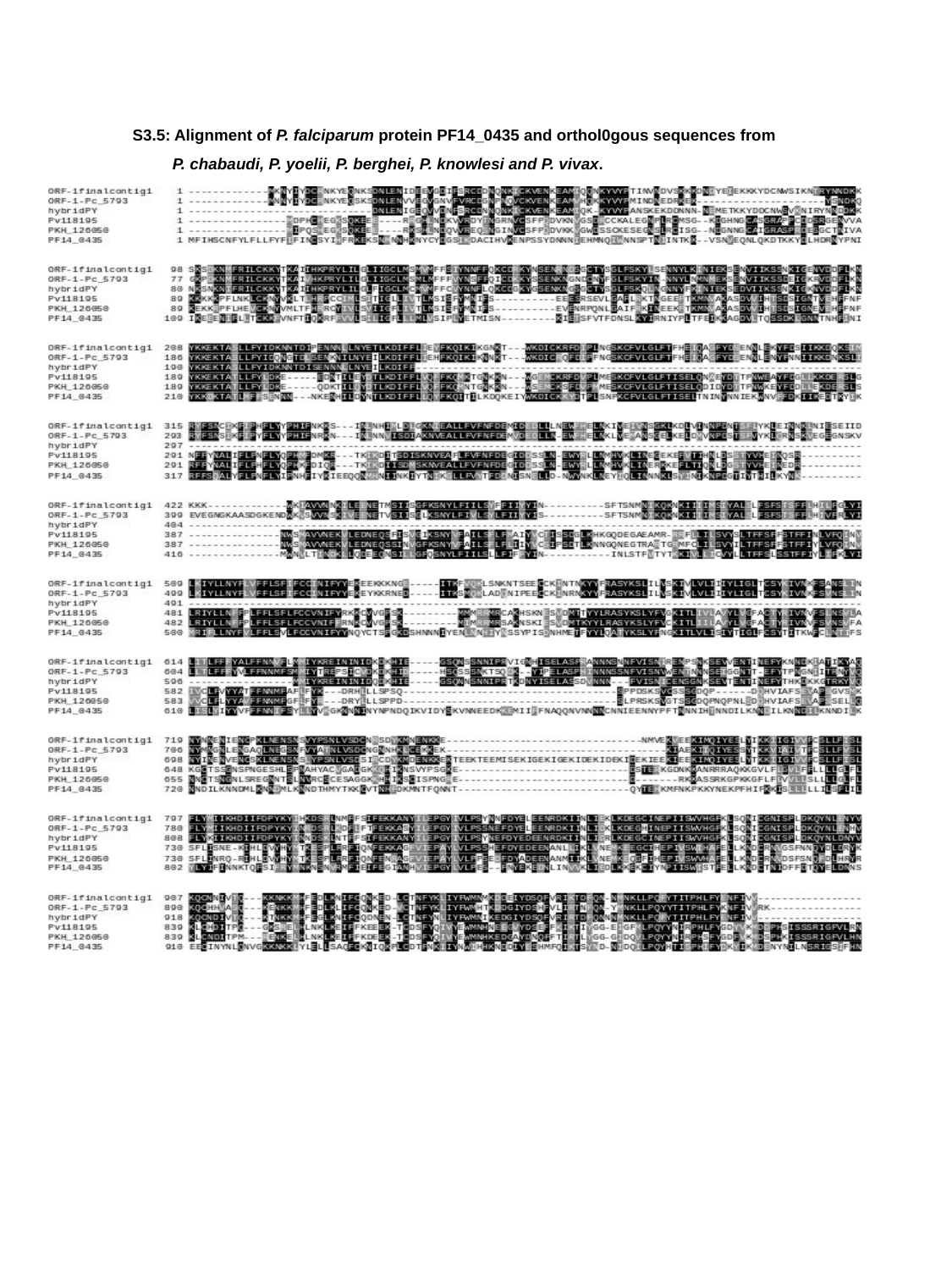

S3.5: Alignment of P. falciparum protein PF14_0435 and orthol0gous sequences from
 P. chabaudi, P. yoelii, P. berghei, P. knowlesi and P. vivax.

## Slide 6
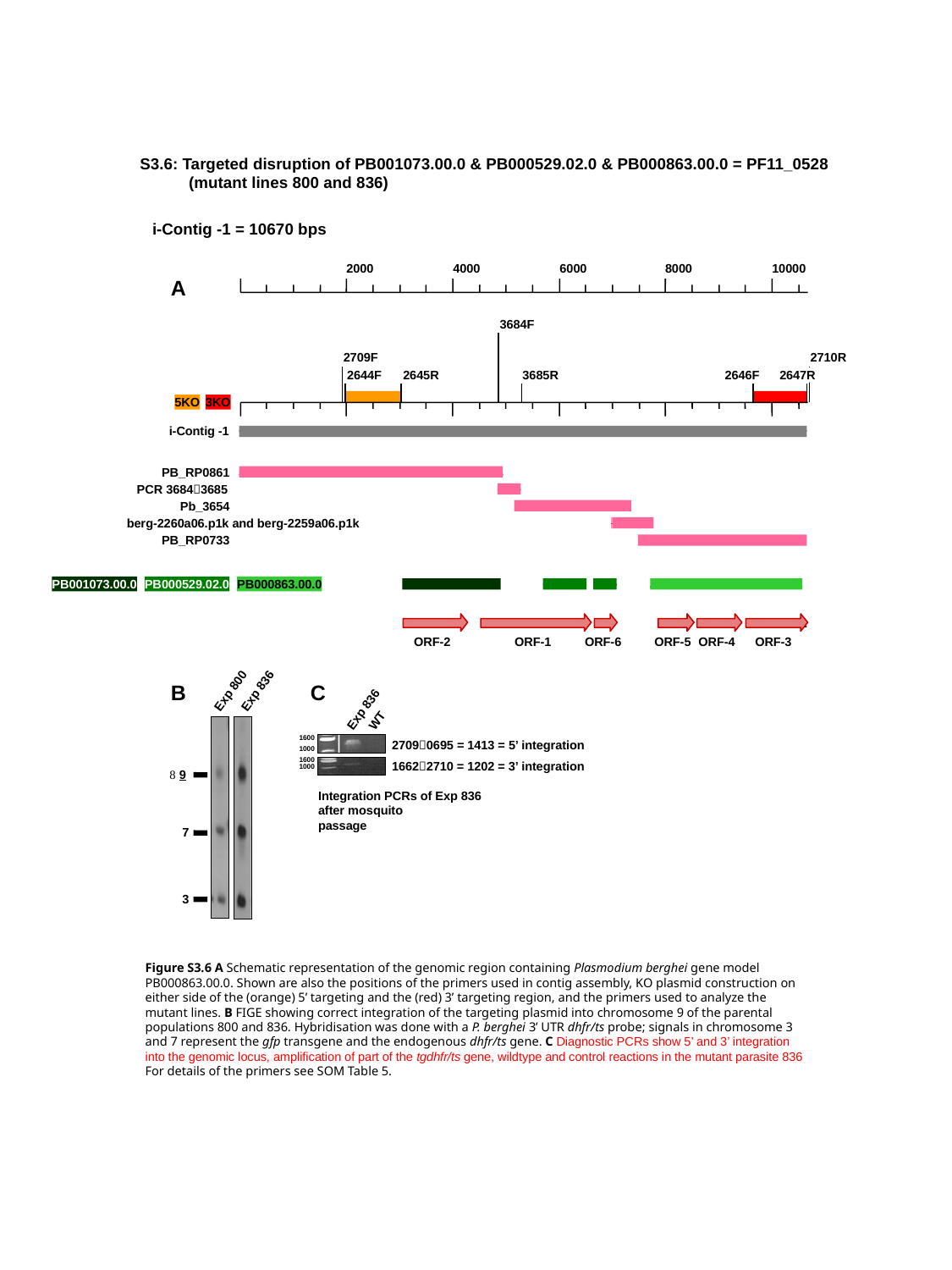

S3.6: Targeted disruption of PB001073.00.0 & PB000529.02.0 & PB000863.00.0 = PF11_0528
 (mutant lines 800 and 836)
i-Contig -1 = 10670 bps
2000
4000
6000
8000
10000
A
3684F
2709F
2710R
2644F
2645R
3685R
2646F
2647R
5KO
3KO
i-Contig -1
PB_RP0861
PCR 36843685
Pb_3654
berg-2260a06.p1k and berg-2259a06.p1k
PB_RP0733
PB001073.00.0
PB000529.02.0
PB000863.00.0
ORF-2
ORF-1
ORF-6
ORF-5
ORF-4
ORF-3
B
C
Exp 836
Exp 800
Exp 836
WT
1600
27090695 = 1413 = 5’ integration
1000
1600
16622710 = 1202 = 3’ integration
1000
 9
Integration PCRs of Exp 836
after mosquito
passage
7
3
Figure S3.6 A Schematic representation of the genomic region containing Plasmodium berghei gene model PB000863.00.0. Shown are also the positions of the primers used in contig assembly, KO plasmid construction on either side of the (orange) 5’ targeting and the (red) 3’ targeting region, and the primers used to analyze the mutant lines. B FIGE showing correct integration of the targeting plasmid into chromosome 9 of the parental populations 800 and 836. Hybridisation was done with a P. berghei 3’ UTR dhfr/ts probe; signals in chromosome 3 and 7 represent the gfp transgene and the endogenous dhfr/ts gene. C Diagnostic PCRs show 5’ and 3’ integration into the genomic locus, amplification of part of the tgdhfr/ts gene, wildtype and control reactions in the mutant parasite 836
For details of the primers see SOM Table 5.

## Slide 7
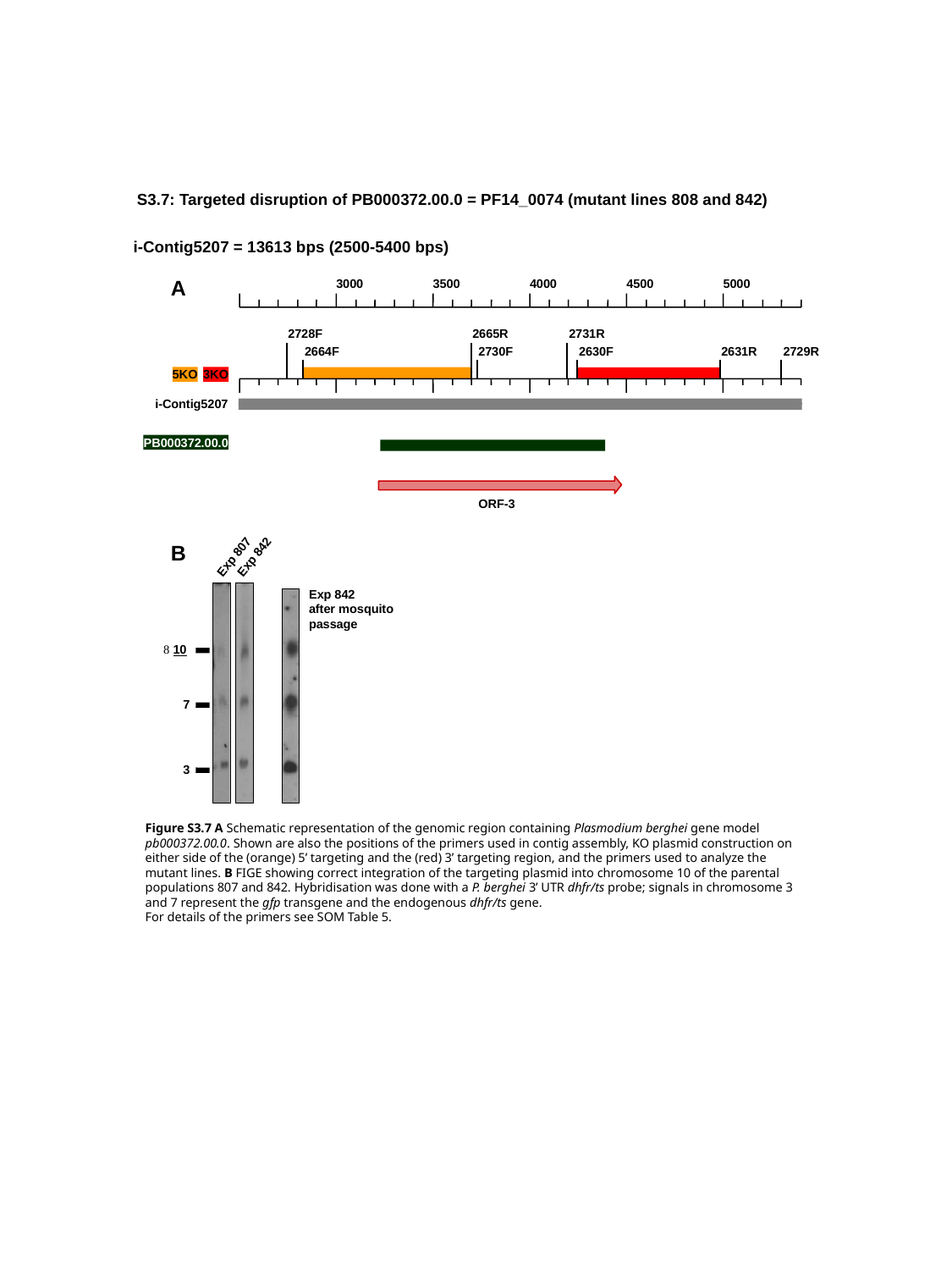

S3.7: Targeted disruption of PB000372.00.0 = PF14_0074 (mutant lines 808 and 842)
i-Contig5207 = 13613 bps (2500-5400 bps)
A
3000
3500
4000
4500
5000
2728F
2665R
2731R
2664F
2730F
2630F
2631R
2729R
5KO
3KO
i-Contig5207
PB000372.00.0
ORF-3
B
Exp 807
Exp 842
Exp 842
after mosquito
passage
 10
7
3
Figure S3.7 A Schematic representation of the genomic region containing Plasmodium berghei gene model pb000372.00.0. Shown are also the positions of the primers used in contig assembly, KO plasmid construction on either side of the (orange) 5’ targeting and the (red) 3’ targeting region, and the primers used to analyze the mutant lines. B FIGE showing correct integration of the targeting plasmid into chromosome 10 of the parental populations 807 and 842. Hybridisation was done with a P. berghei 3’ UTR dhfr/ts probe; signals in chromosome 3 and 7 represent the gfp transgene and the endogenous dhfr/ts gene.
For details of the primers see SOM Table 5.

## Slide 8
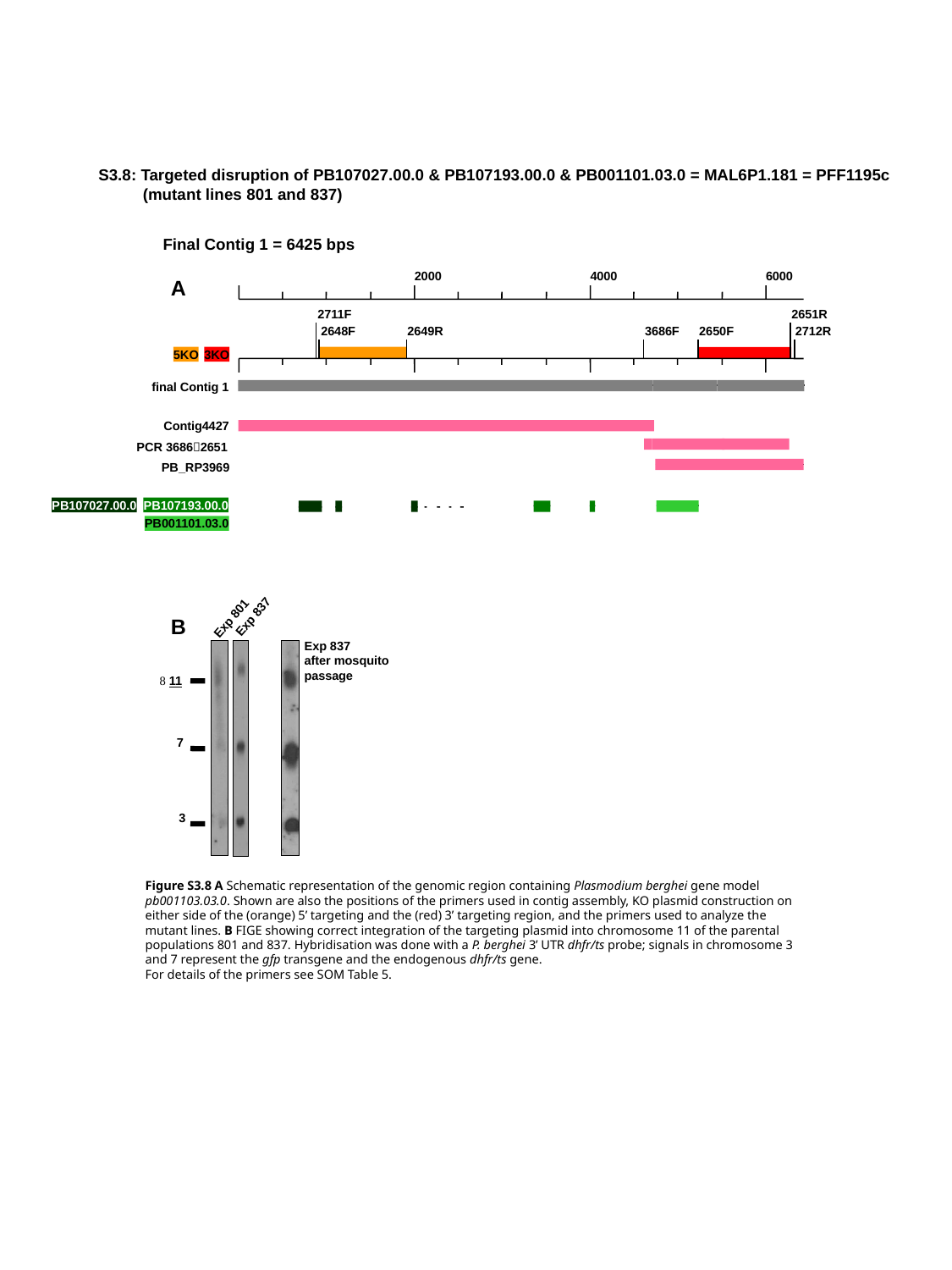

S3.8: Targeted disruption of PB107027.00.0 & PB107193.00.0 & PB001101.03.0 = MAL6P1.181 = PFF1195c
 (mutant lines 801 and 837)
Final Contig 1 = 6425 bps
2000
4000
6000
2711F
2651R
2648F
2649R
3686F
2650F
2712R
5KO
3KO
final Contig 1
Contig4427
PCR 36862651
PB_RP3969
PB107027.00.0
PB107193.00.0
PB001101.03.0
A
Exp 837
Exp 801
B
Exp 837
after mosquito
passage
 11
7
3
Figure S3.8 A Schematic representation of the genomic region containing Plasmodium berghei gene model pb001103.03.0. Shown are also the positions of the primers used in contig assembly, KO plasmid construction on either side of the (orange) 5’ targeting and the (red) 3’ targeting region, and the primers used to analyze the mutant lines. B FIGE showing correct integration of the targeting plasmid into chromosome 11 of the parental populations 801 and 837. Hybridisation was done with a P. berghei 3’ UTR dhfr/ts probe; signals in chromosome 3 and 7 represent the gfp transgene and the endogenous dhfr/ts gene.
For details of the primers see SOM Table 5.

## Slide 9
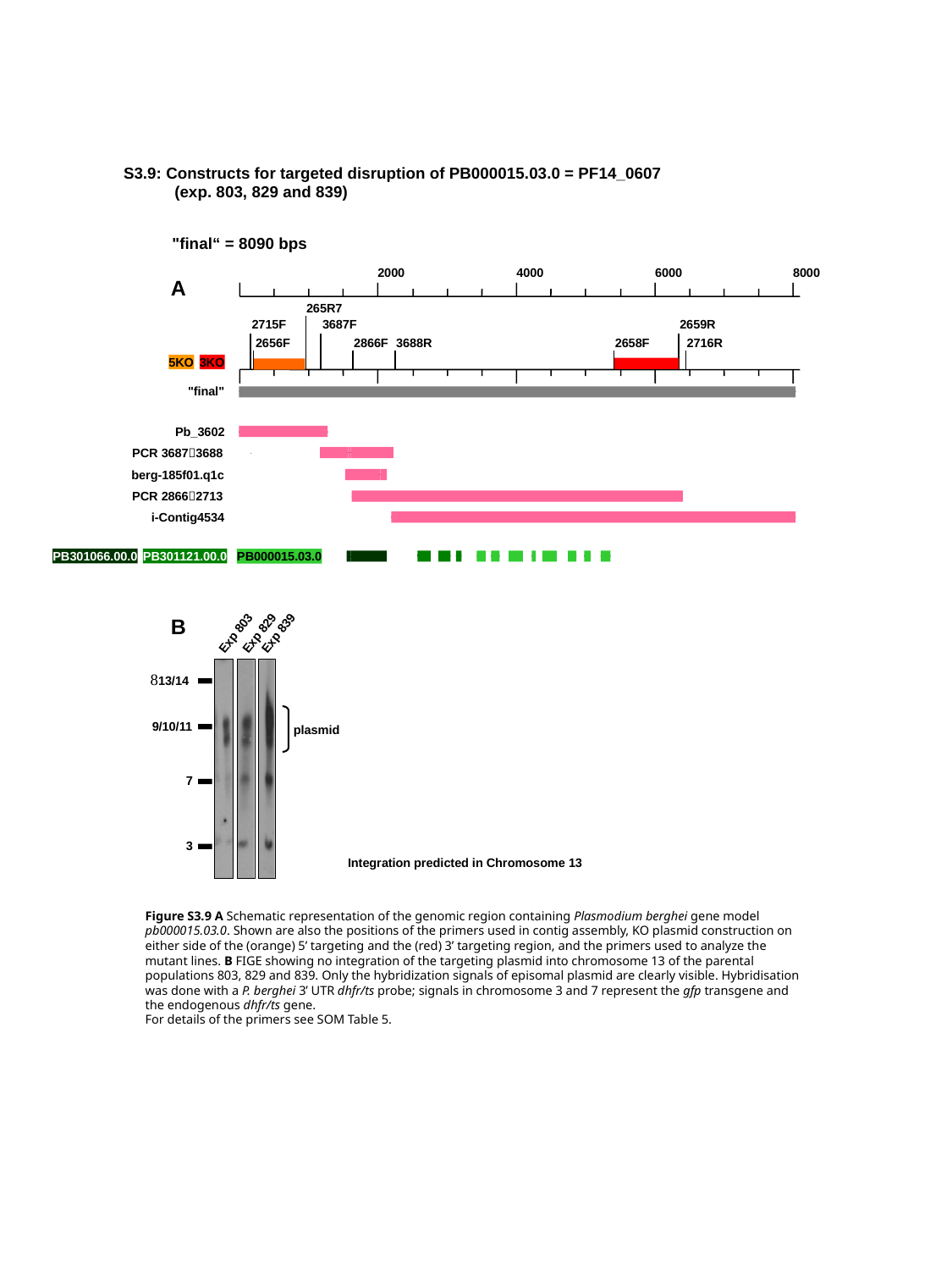

S3.9: Constructs for targeted disruption of PB000015.03.0 = PF14_0607
 (exp. 803, 829 and 839)
"final“ = 8090 bps
2000
4000
6000
8000
265R7
2715F
3687F
2659R
2656F
2866F
3688R
2658F
2716R
5KO
3KO
"final"
Pb_3602
PCR 36873688
berg-185f01.q1c
PCR 28662713
i-Contig4534
PB301066.00.0
PB301121.00.0
PB000015.03.0
A
B
Exp 803
Exp 829
Exp 839
13/14
9/10/11
plasmid
7
3
Integration predicted in Chromosome 13
Figure S3.9 A Schematic representation of the genomic region containing Plasmodium berghei gene model pb000015.03.0. Shown are also the positions of the primers used in contig assembly, KO plasmid construction on either side of the (orange) 5’ targeting and the (red) 3’ targeting region, and the primers used to analyze the mutant lines. B FIGE showing no integration of the targeting plasmid into chromosome 13 of the parental populations 803, 829 and 839. Only the hybridization signals of episomal plasmid are clearly visible. Hybridisation was done with a P. berghei 3’ UTR dhfr/ts probe; signals in chromosome 3 and 7 represent the gfp transgene and the endogenous dhfr/ts gene.
For details of the primers see SOM Table 5.

## Slide 10
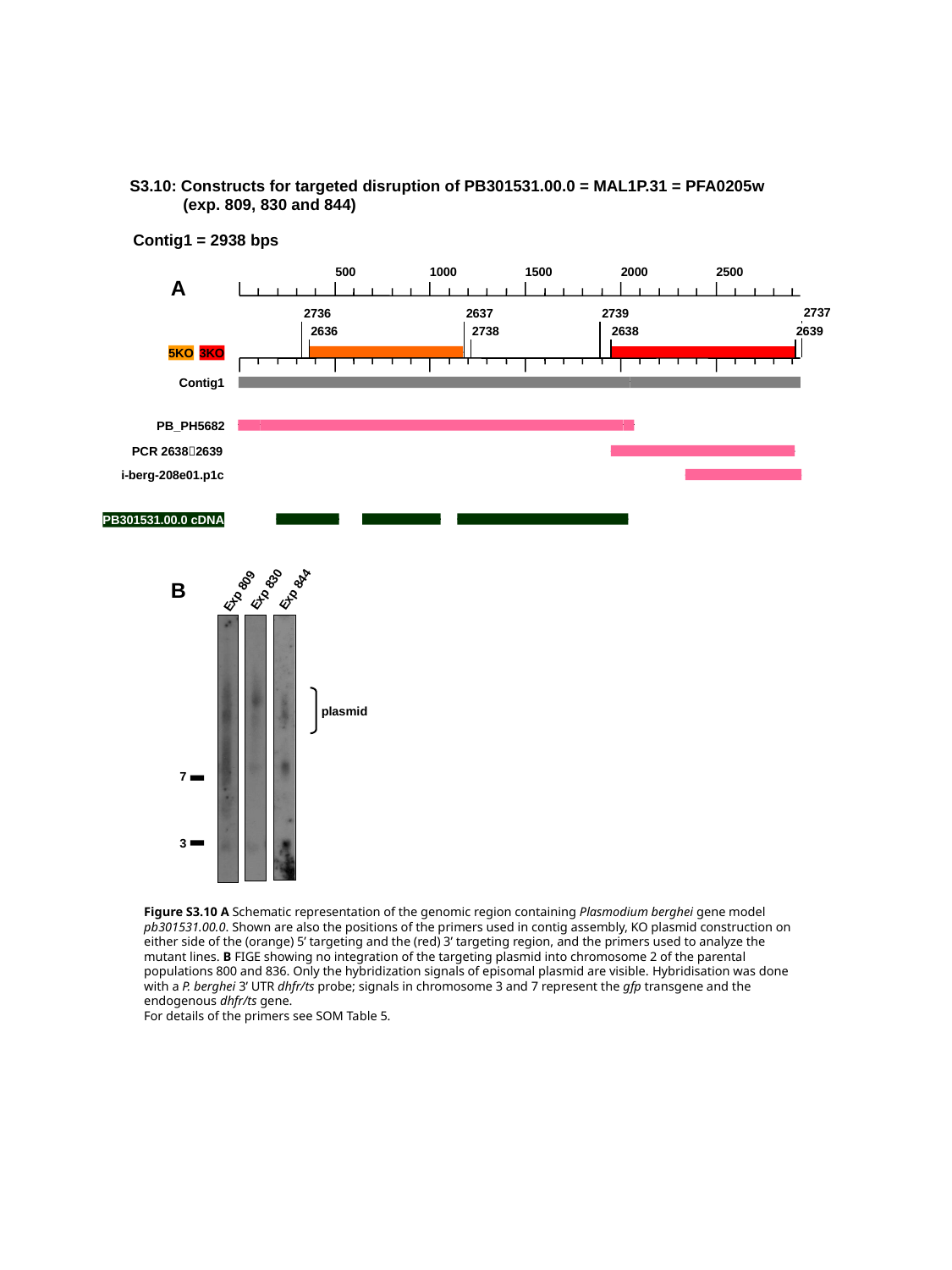

S3.10: Constructs for targeted disruption of PB301531.00.0 = MAL1P.31 = PFA0205w
 (exp. 809, 830 and 844)
Contig1 = 2938 bps
500
1000
1500
2000
2500
2737
2736
2739
2637
2636
2738
2638
2639
5KO
3KO
Contig1
PB_PH5682
PCR 26382639
i-berg-208e01.p1c
PB301531.00.0 cDNA
A
B
Exp 830
Exp 844
Exp 809
plasmid
7
3
Figure S3.10 A Schematic representation of the genomic region containing Plasmodium berghei gene model pb301531.00.0. Shown are also the positions of the primers used in contig assembly, KO plasmid construction on either side of the (orange) 5’ targeting and the (red) 3’ targeting region, and the primers used to analyze the mutant lines. B FIGE showing no integration of the targeting plasmid into chromosome 2 of the parental populations 800 and 836. Only the hybridization signals of episomal plasmid are visible. Hybridisation was done with a P. berghei 3’ UTR dhfr/ts probe; signals in chromosome 3 and 7 represent the gfp transgene and the endogenous dhfr/ts gene.
For details of the primers see SOM Table 5.

## Slide 11
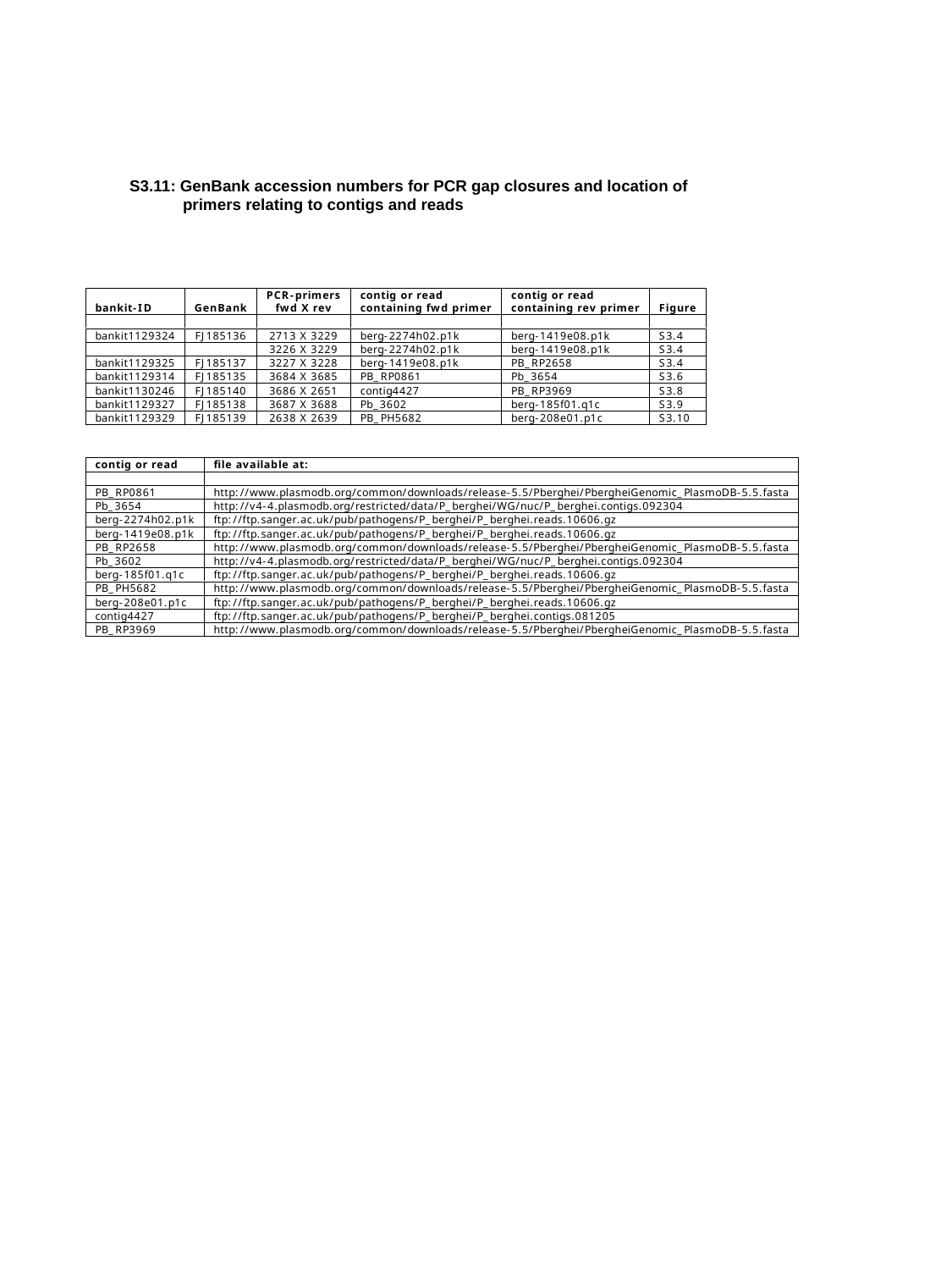

S3.11: GenBank accession numbers for PCR gap closures and location of
 primers relating to contigs and reads
